# Impact of inundation range of overtopping dam break of tailings pond under actual terrain conditions

**DOI:** 10.1371/journal.pone.0295056

**Published:** 2023-12-06

**Authors:** Changbo Du, Ben Niu, Fu Yi, Xinqi Jiang, Lidong Liang

**Affiliations:** 1 School of Civil Engineering, Liaoning Technical University, Fuxin, Liaoning Province, China; 2 Beijing Jingneng Geological Engineering Co.,Ltd, Beijing, China; ICIMOD: International Centre for Integrated Mountain Development, NEPAL

## Abstract

Tailing ponds are a major hazard source with the risk of dam breaks. To predict the impact of tailings pond dam breaks more accurately, one needs to quantitatively understand the dam-breaking process of the tailings reservoir and its downstream impact. This study is based on an old tailings pond that is about to be put out of service and the proposed new tailings pond next to it. Study the inundation range of the new and old tailing ponds with simultaneous overtopping dam breaks under actual terrain conditions. First, fine-grained tailings and expanded perlite were selected as the model sand materials, and the appropriate model sand ratio was determined through laboratory tests. Second, the two tailings ponds were tested (at a scale of 1:200), for flood overtopping and simultaneous dam breaks. The dam break, flow, section morphology evolution, submerged elevation, and range were analyzed. Finally, a numerical model was developed using MIKE 21 to simulate the simultaneous overtopping and collapse of the new and old tailings ponds, and the impact of rainfall intensity on the inundation range of the simultaneous overtopping of the dam was analyzed. The research results will guide disaster prevention and mitigation in tailings reservoirs.

## 1 Introduction

Tailings ponds are a critical component of safety supervision. Once a dam breaks, the destructive water-sand mixture rushes downstream, causing severe environmental pollution and posing great harm to the lives and property of people in the downstream areas. Dam-break causes include flood overtopping, seepage breaks, seismic liquefaction, excessive saturation lines, and excessive rising speed of the dam body [1]. There are many reasons for tailings dam breaks in China and abroad, such as earthquakes [2], though the proportion caused by flood overtopping is the highest. Consequently, it is important to conduct an overtopping dam-break test of the tailings pond and analyze the dam-break process and its influence downstream.

According to the State Administration of Safety Supervision requirements, most tailings-pond areas require dam-break simulation tests to evaluate their safety [3]. To quantify flood disasters associated with dam failures, flood variables must be predicted using efficient and robust numerical models. These models can effectively address the computational difficulties caused by complex variables and terrain [4]. Many scholars have used models to study tailings dam breaks. Wang *et al*. [5] extended the one-dimensional finite volume model to a two-dimensional model, successfully validating the two-dimensional model through three analysis benchmark tests, before evaluating the prediction of actual dam-break flood events. Petaccia [6] proposed two two-dimensional parallel dam-break models based on the shallow water equation and applied them to laboratory testing and natural dam breaks. Issakhov *et al*. [7] conducted accuracy and reliability tests on two-dimensional and three-dimensional models of dam breaks using multiple small and large laboratory experiments; the numerical results were consistent with the experimental results. Chang *et al*. [8–13] conducted a series of dam-break studies using smooth-particle hydrodynamics. Li [14] proposed a new double-layer-averaged model that was more consistent with experimental measurements of instantaneous and progressive dam-break floods. Seyedashraf O [15] proposed and tested a new method based on a computational intelligence system to simulate the classical one-dimensional dam-break flow problem. Evangelista S [16] developed a multistage first-order central scheme GMUSTA to simulate dam-break floods with and without sediment transport. Liu [17] designed a physical model of a tailings pond using the similarity model theory and observed the evolution of the discharged flood. Qi *et al*. [18, 19] conducted a physical test on the break of a drainage system, which led to the continuous rise of the phreatic line and induced dam break, summarizing the modes and phenomena of damage caused by the drainage break. Yin *et al*. [20] studied the evolution law and dynamic characteristics of debris flow after a tailings dam breakthrough using a similar simulation test. Wang *et al*. [21] analyzed the mechanism of an overtopping dam break under rainfall and the change of the phreatic line during rain using model tests. The results showed that the water level of the tailings pond greatly affected the phreatic line during rain. Zhang *et al*. [22] used a dam displacement tracking system to conduct model tests on the dam break of tailings ponds caused by seepage breaks and summarized its mode and process. Kong *et al*. [23] conducted tests on the dam-break mode of a tailings pond under the condition of pond water level fluctuation, obtained the evolution factors of the tailings dam break, and analyzed the displacement of the dam body after the dam break. Dang and Gao *et al*. [24] conducted dam-break tests on the flood overtopping of tailing ponds under different levels of compactness. Analysis of the key damage nodes showed that increasing the stacking compactness of the tailings dam could effectively delay the formation time of the debris flow. To quantitatively grasp the dam-break process of the tailings pond and its downstream impact, Sun *et al*. [25] established a tailings-pond model based on the similarity model theory, recorded the dam-break process in detail, and analyzed the dam site flow, water level process line, and water-sand flow evolution law. Yang *et al*. [26–28] combined MIKE21 with different models to establish a new model to study the dam-break problem. Yang *et al*. [29] used a mathematical model and Fluent software to establish the model and found that the actual terrain greatly influenced the dam breakage of the tailings pond.

In summary, many scholars have used physical model tests and numerical simulations to analyze the downstream evolution process of tailings flow, downstream water levels, and the formation and development of breaches. However, most model tests on dam breaks are small and cannot adequately reflect the three-dimensional effect, making further analysis of large-scale model tests essential. Large-scale studies can more accurately restore the process of on-site dam breaks more accurately. In this study, using a tailings pond about to be put out of service and a planned tailings pond adjacent to it as the engineering background (referred to as the old pond and the new pond, respectively), large-scale scaling of the tailings pond was conducted. Moreover, tests were conducted on the simultaneous overtopping of the new and old tailings ponds under actual terrain conditions, and numerical simulations were used to compare and verify the results, the results providing suggestions for the relocation of downstream residents and structures.

## 2 Overtopping dam-break model test design and model sand ratio selection

### 2.1 Project overview

The final stacking elevation of the old tailings pond design was 452 m, the total dam height was 172.2 m, and the full storage capacity was 6970.7 × 104 m^3^, making it a second-class pond. As of April 2020, the old pond had risen to an elevation of 434.5 m, and the remaining storage capacity was expected to serve only until the beginning of 2022. The subsequent tailing storage problem was a key factor restricting the subsequent development of mines. To meet the production and development needs of mines, we proposed a new tailings pond adjacent to the old pond. The total dam height of the new pond was 175.2 m, the full storage capacity being 30.76 million m^3^.

**Fig 1:**
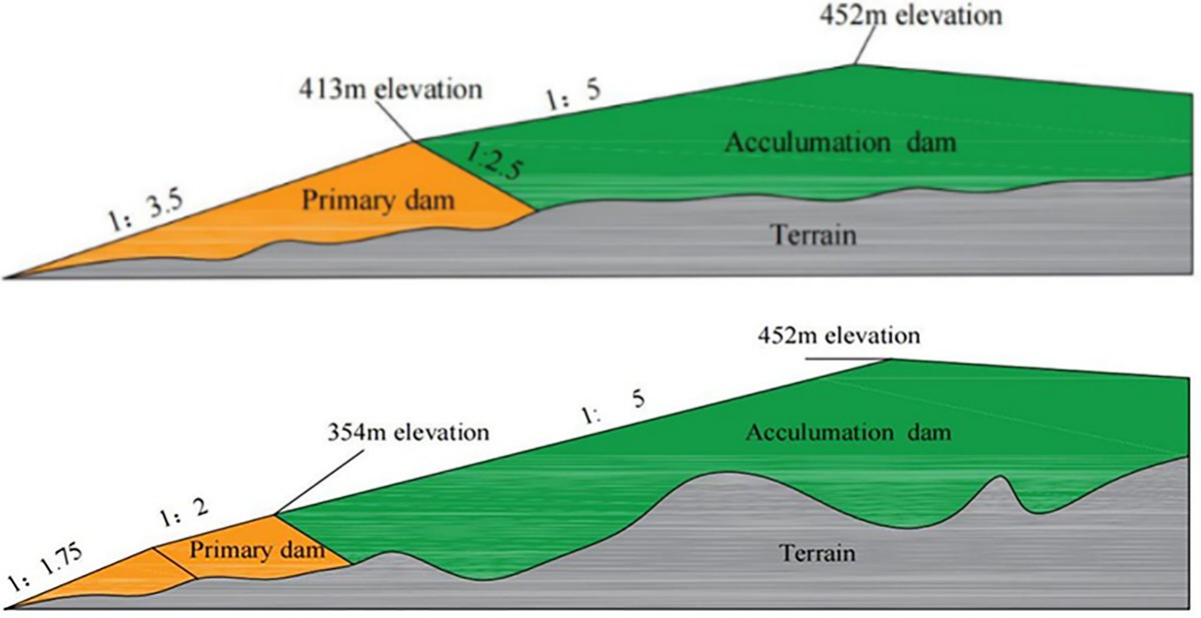
Tailings reservoir profile. (a) New tailings pond profile, (b) Old tailings pond profile.

**Fig 2:**
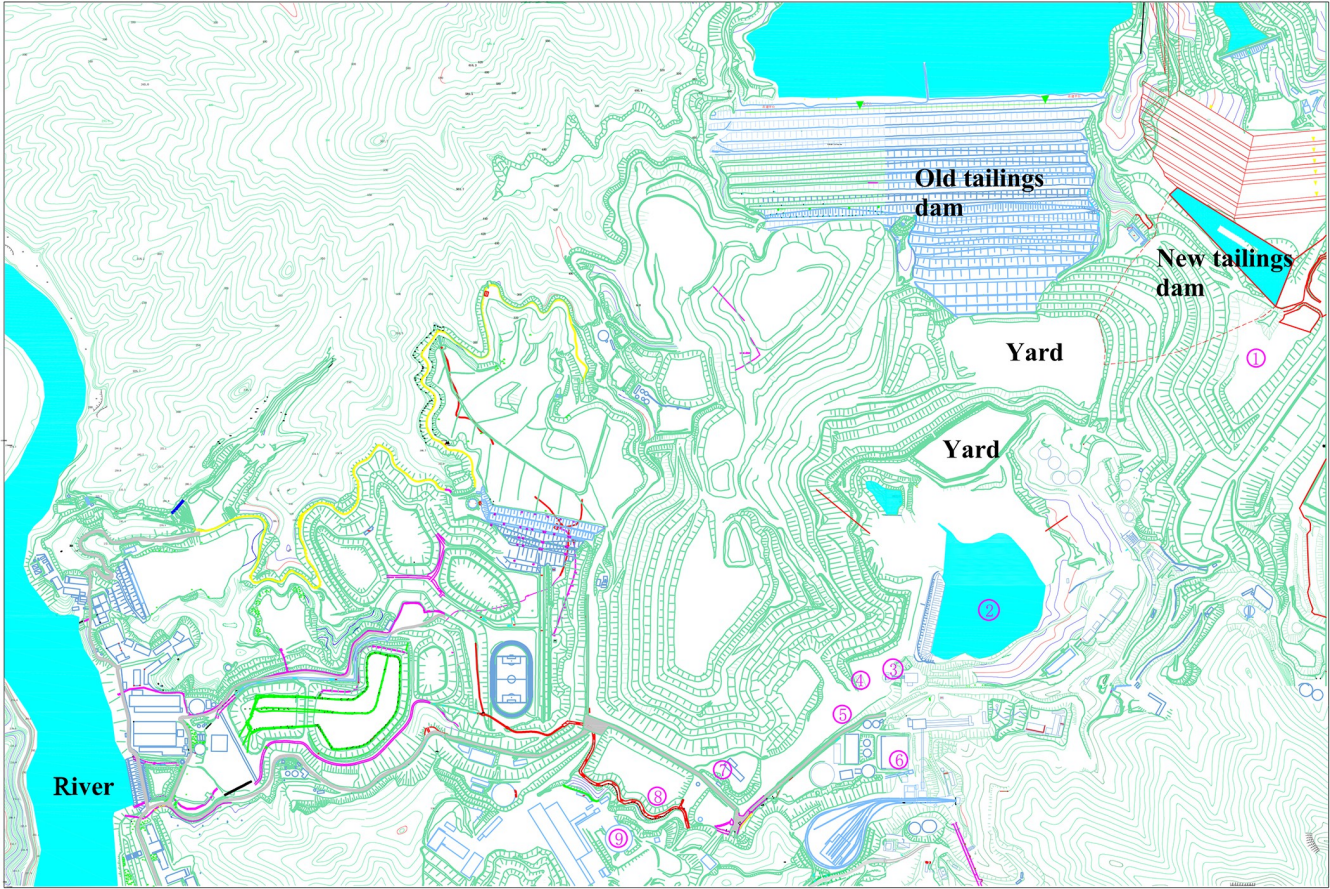
Drawing of the downstream facilities of the tailings pond.

A specific overview of the facilities in the areas surrounding the new and old tailing ponds is provided in **Table 1**. The tailings pond profiles are shown in **Fig 1**. A schematic of the location relationship is shown in **Fig 2**.

**Table 1:** Inundation impact range.

Location point	Elevation (m)	Location point	Elevation (m)	Location point	Elevation (m)
① Repair shop	350 m	② Reservoir	258.9 m	③ Construction	280.0 m
④ Repair shop	300.0 m	⑤ Road	308.0 m	⑥ Concentrator	320 m
⑦ Construction	330.0 m	⑧ Warehouse	239.0 m	⑨ Construction	232.0 m

### 2.2 Model sand selection

To successfully conduct simulations, selecting a suitable sand material for simulation is a crucial step. A mixture of fine-grained tailings and expanded perlite from the tailings pond was initially selected as the model sand material for the physical model test. The model sand had a moderate bulk density, stable chemical properties, and was homologous to the materials used in the dam construction. Its physical and mechanical properties were similar. Moreover, the particle size of the fine-grained tailings in the pond was relatively small, making it easy to classify.

**Fig 3:**
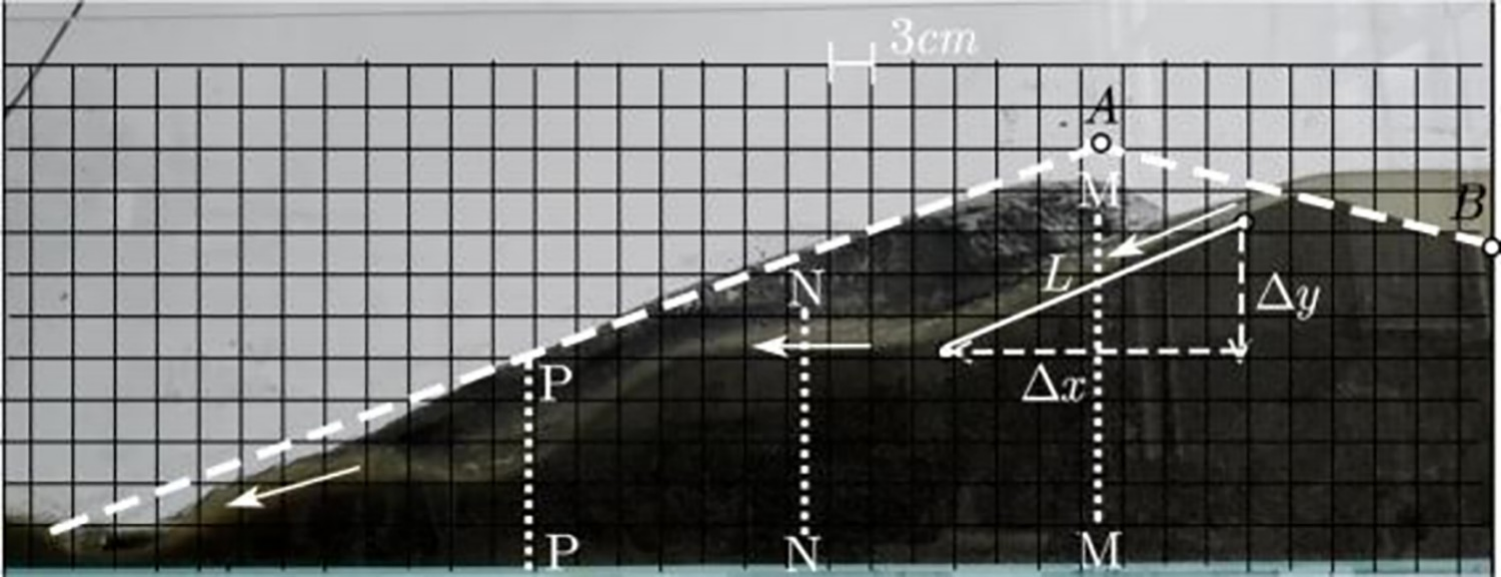
The sink test.

To facilitate the test and save materials, the flume test was used to explore the ratio of model sand materials, conduct a flume test based on the average outer slope ratio of the actual accumulation dam, and stack the model sand in the flume. When piling the model sand, pressing, and tamping was performed to avoid piping failure. After completing the model, water was added to the pond using a container to simulate an overtopping break in the dam body caused by local rainfall. The dimensions of the water tank were 3000 × 300 × 500 mm (length × width × height), as shown in Fig 3. Based on the data obtained from the indoor flume test and factors such as the dam-break shape and sand-carrying capacity of the model sand with each ratio during the test, the model sand with the ratio of fine-grained tailings to expanded perlite of 1.5:1 had the best effect. At this point, we could obtain *λv*_c_ = 13.5~14.2 using the corresponding starting velocity of the model sand and prototype sand, close to the velocity scale 14, to ensure the motion similarity of the model sand. The specific gravity of the model sand *γ*_s_ = 2.0, proportion of the prototype sand *γ*_s_ = 2.9, and bulk density scale *λ*_*γ*_ = 1.45 were used to further determine a selected model sand that could approximately satisfy the starting similarity conditions.

**Fig 4:**
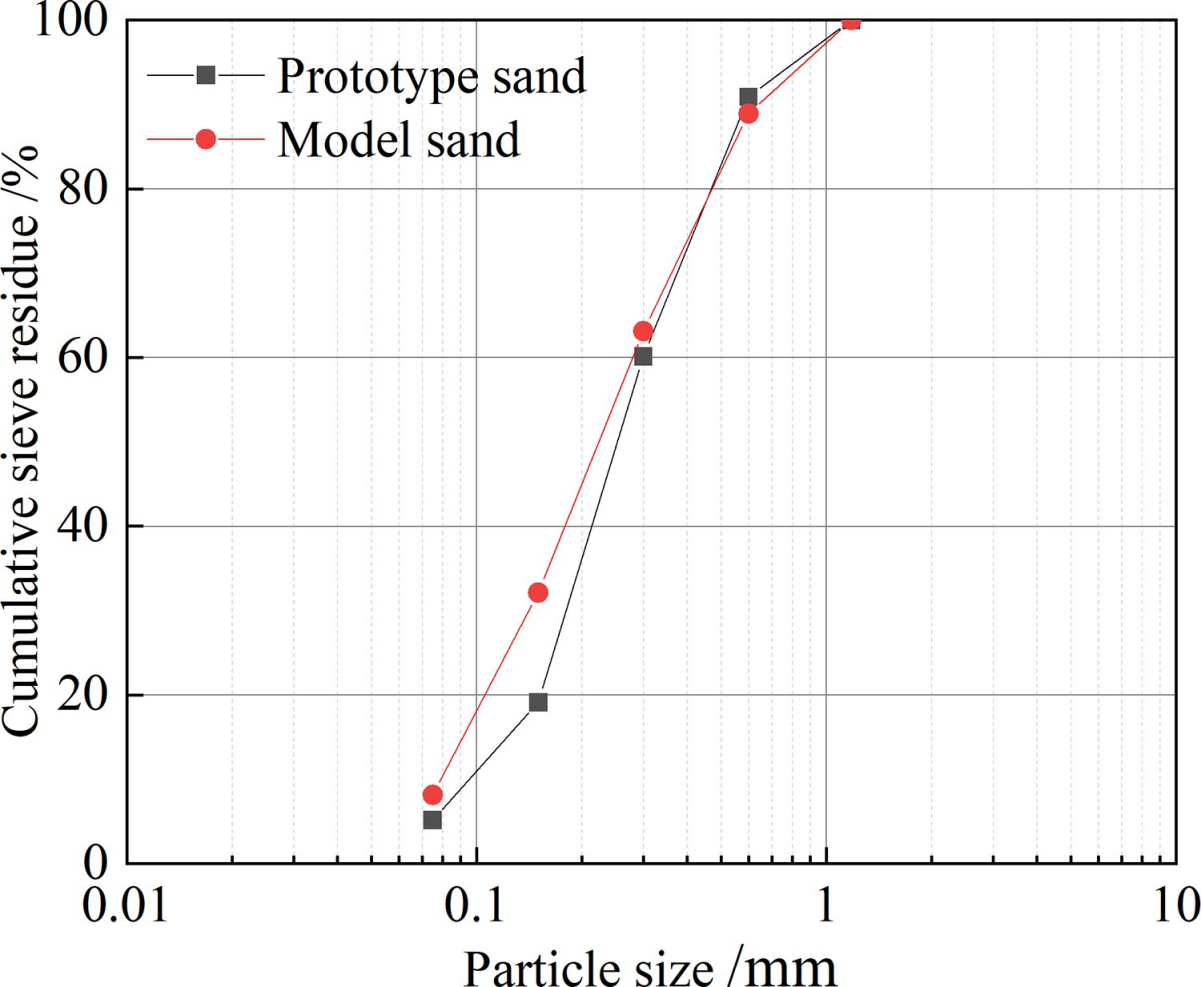
Grading curve.

The grading curves of the prototype and model sands are shown in **Fig 4**. The non-uniformity coefficient of the prototype sand *C*_u_ = 3 < 5 and the curvature coefficient *C*_c_ = 1.3 is between 1 and 3, indicating that the prototype sand is poorly graded. The nonuniformity coefficient of the model sand *C*_u_ = 4 < 5 and the curvature coefficient *C*_c_ = 0.952 < 1 indicate that the model sand has poor gradation; however, the *C*_u_ of the model sand is greater than that of the prototype sand, and the particle gradation of the model sand is more uniform.

Using this ratio of model sand for routine physical and mechanical tests, the physical and mechanical properties of the model sand and prototype sand were compared, as shown in **Table 2**. The particle size distribution curve and permeability performance of the model sand and prototype sand are similar, the difference in cohesion and friction angle between the two not being significant, indicating that the physical and mechanical properties of the selected model sand are similar to those of the prototype sand, ensuring the similarity of the collapse displacements at the two breaches.

**Table 2:** Summary of the physical and mechanical indexes of the prototype sand and model sand.

Physical and mechanical indexes	Particle density (g·cm^3^)	Void ratio	Permeability coefficient (cm·s^-1^)	Cohesive force (kPa)	Internal friction angle (°)
Prototype sand	2.76	0.824	8.167 × 10^−4^	18.35	27.02
Model sand	1.90	0.9	6.12 × 10^−5^	8.68	28.36

The bulk density of the crushed foam brick used in this test as the model material for the primary dam was 1.4. In the preliminary preparation stage, crushed foam bricks of particle size 2–5 mm were used. Finally, it was reasonable to use them in the simulation tests.

### 2.3 Model similarity scale

Based on the size of the test site and the actual engineering situation, the horizontal scale was determined (*λ*_*L*_ = 200), the model test being a standard model to accurately describe the general situation of the project, and make the dam-break process of the tailings pond more realistic. The major scales, based on the similarity theory conditions [14], are shown in **Table 3**.

**Table 3:** Summary of the dam-break model scale of tailings pond.

Similar condition	Scale name	Numerical value	Explanation
Geometric similarity	Horizontal scale *λ*_*L*_	200	Combined with the original data and test conditions to determine
	Vertical scale *λ*_*H*_	200	normal model: *λ*_*L*_ = *λ*_*H*_
	Area scale *λ*_*A*_	40,000	$\lambda_{A}=\lambda_{L}^{2}$
	Volume scale *λ*_*VT*_	8,000,000	$\lambda_{VT}=\lambda_{L}^{2}\lambda_{H}$
Time similar	Time scale *λ*	14	*λ*_*t*_ = *λ*_*L*_/*λ*_*V*_
Fluid motion similarity	Velocity scale *λ*_*V*_	14	$\lambda_{V}=\sqrt{\lambda_{H}}$
	Discharge scale ratio *λ*_*Q*_	565,685	*λ*_*Q*_ = *λ*_*V*_*λ*_*H*_*λ*_*L*_
Similar movement of tailings	Bulk density scale *λ*_*γ*_	1.45	According to the prototype tailings and model sand obtained
	Sand content scale *λ*_*S*_	1.5	
	Grain-size scale *λ*_*d*_	5.6	
	Starting velocity similarity scale *λ*_*VC*_	14	$\lambda_{VC}=\sqrt{\lambda_{H}}$ and flume test

### 2.4 Establishing a physical model

**Fig 5:**
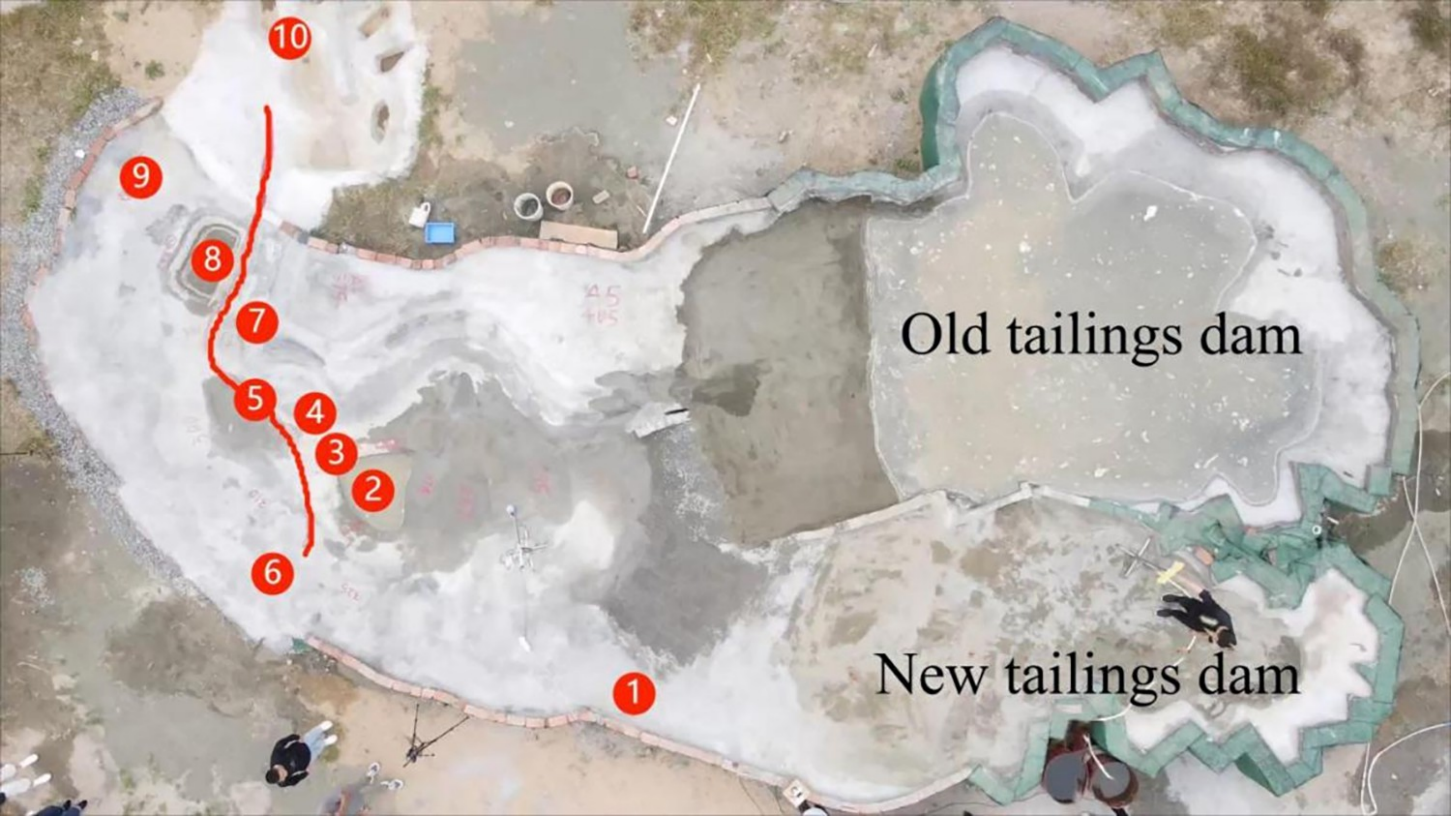
Downstream layout of the tailings pond.

The actual terrain projection area simulated in this study was (1440 × 720) m^2^. Based on the scale of 1:200, the projection area of the physical model made was 7.2 × 3.6 m^2^, including the main part of the tailings pond, the terrain and structures in the downstream channel and downstream range, the reservoir, the rainfall system, and the tailwater collection system. The tailings pond and downstream terrain were built into fixed beds, and the scoured tailings dam was built with model sand. The model sand was prepared based on the ratio of the model sand, and the internal and downstream structures of the two tailing ponds were constructed, as shown in **Fig 5**.

## 3 Model test research of overtopping dam break of tailings pond

### 3.1 Cross-section selection and testing measurement equipment

**Fig 6:**
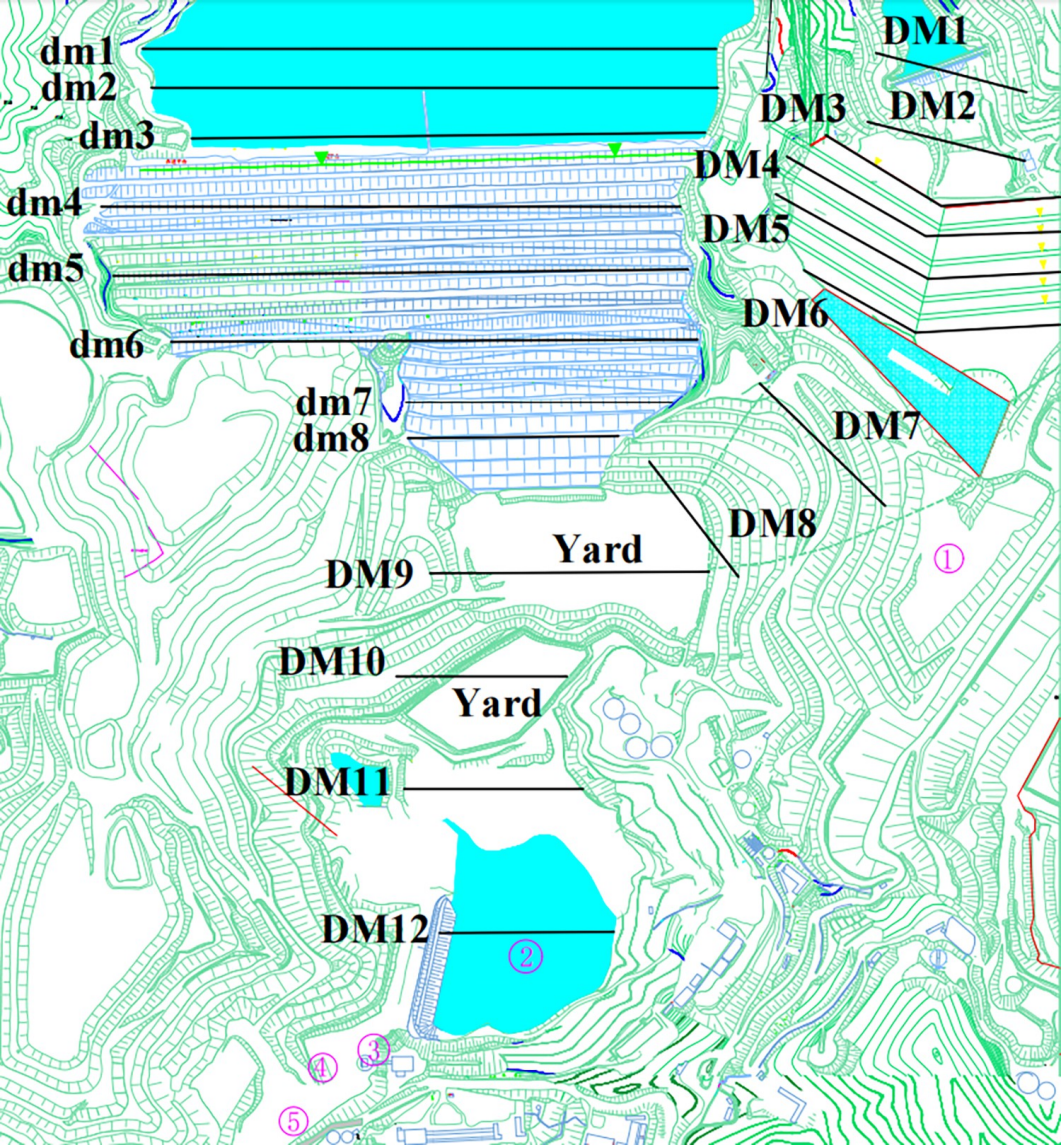
Location of each measurement section.

To better quantitatively analyze the process of simultaneous overtopping and dam breakage of the two tailings ponds, 20 detection sections were set up in the tailings pond, primary dam, accumulation dam, and downstream of the two tailings ponds—that is, **dm1–dm8** of the old pond, **DM1–DM8** of the new pond, and **DM9–DM12** of the downstream section. The closest **DM9** section was located at the primary dam site and the farthest **DM12** section was approximately 610 m from the primary dam site of the tailings pond. Specific information on the monitoring sections is shown in **Table 4**, and a distribution diagram of the monitoring sections is shown in **Fig 6**.

**Table 4:** Summary table of monitoring section information.

Location of the section	Section name	Section elevation (m)	Location of the section	Section name	Section elevation (m)
The old tailings pond	dm1	450	The new tailings pond	DM1	450
	dm2	451		DM2	451
Old pond accumulation dam	dm3	452	New pond accumulation dam	DM3	452
	dm4	427		DM4	442
	dm5	402		DM5	432
	dm6	377		DM6	422
Old pond primary dam	dm7	354	New pond primary dam	DM7	413
	dm8	324		DM8	390
Downstream	DM9	316			
	DM10	287			
	DM11	274			
	DM12	260			

**Fig 7:**
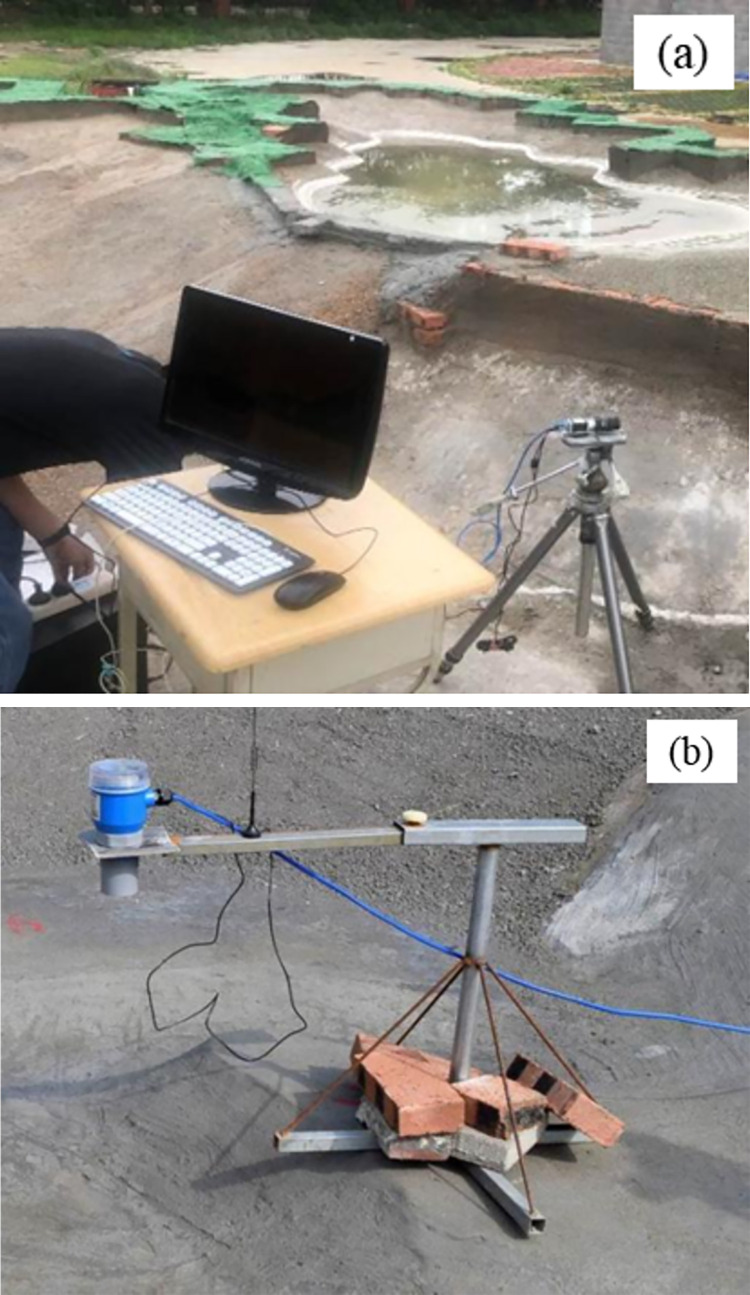
Test equipment: (a) HD camera monitoring system; (b) Ultrasonic liquid level meter.

The model test equipment included a saturation line monitoring system, flow change monitoring equipment, water level change line monitoring equipment, and section monitoring equipment. Saturation line monitoring was performed by converting the pore-water pressure meter data into a pressure head and adding it to the location head to obtain the total head height. This system serves to control the location of the saturation line of the model dam to be the same as the prototype dam. The flow monitoring using a drone was at close range. The flow of the tracer calculated the flow rate, after which the flow change could be obtained. Water level change line monitoring was used to measure the water level depth in real time using an ultrasonic liquid level meter to record and monitor the shape of the cross-section and the process of dam failure. Finally, high-definition camera monitoring systems were used to monitor real-time changes in the cross-section. The monitoring equipment was as shown in **Fig 7**.

### 3.2 Rainfall flow calculation of the model

The rainfall system comprised a water storage facility, atomizing nozzle, tee, and water pipe. The total rainfall flow could be comprehensively calculated using the total volume of water required for the rainfall to calculate the rainfall intensity. Based on the Code for Design of Tailings Facilities (GB 50863–2013), tailings pond flood control and drainage standards are implemented once every 1000 years (P = 0.1%). Consequently, the dam-break test should primarily simulate the dam-break form when the flood discharge system of the tailings pond loses its flood discharge capacity in the extreme case of maximum sudden rainstorm flood overtopping. Based on the preliminary results, when the flood prevention and drainage standard of the tailings pond was set to once every 1000 years (P = 0.1%), the rainfall depth of the model dam at 60 min was approximately 2.145 mm, and the submerged dry beach length of the model dam was 23.8 cm. Even if a tailings pond flood drainage system lost its flood discharge capacity, dam-break overtopping could not occur. In this study, the simulated rainfall intensity was 2.35 mm/min. Under the failure of the flood discharge system of the tailings pond under this rainfall intensity, an overtopping dam break occurred in the new pond after approximately 13.2 min.

### 3.3 Analysis of the test results of overtopping dam break

After the construction of the physical model, considering the large storage capacity of the two tailings ponds, the water level of the pond reached the maximum safe elevation at the start (to shorten the test time). A camera was placed at each monitoring angle, and the drone was readied. Each piece of the monitoring equipment was placed in its designated position.

#### 3.3.1 Overview of the dam-break process

**Fig 8:**
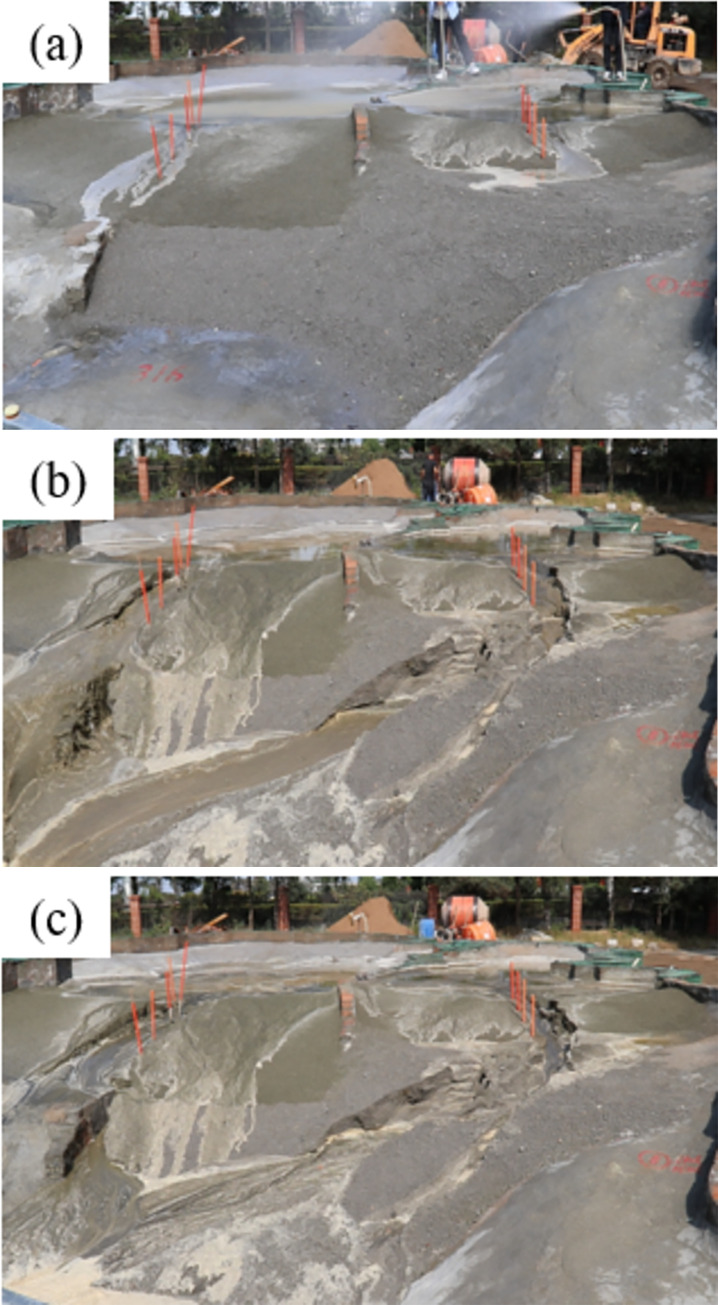
The process of a simultaneous overtopping dam break of the old and new tailings ponds: (a) Early stage; (b) Mid-term; (c) Late stage.

As shown in **Fig 8**, the entire dam-break process can be divided into three parts: early, middle, and late dam breaks.

1. After the start of rainfall, the slope of the dam is constantly scoured. After overtopping of the water flow, the water continues to flow over the surface of the dam body. After 13.2 min of overtopping, the water flow increases and part of the water flow passes through and forms a fixed circulation path. First, a breach of the dam body of the new pond accumulation dam appears. After 26.5 min of overflow, the old pond accumulation dam begins to breach. When the old pond breach is completely formed, a long, thin ditch appears on the slope of the new pond. The tailings rapidly flow down with the local drainage ditch, and the drainage ditch becomes deeper, forming a clear and penetrating drainage ditch. At this time, the sand content in the tailing flow is high.
2. With the progress of the test, owing to the increasing intensity of water erosion, the breach depth of the two tailings dams deepens. The tailings sand flow erodes the area around the breach which gradually became unstable. After overtopping for 36 min, the body of the new pond accumulation dam begins to collapse. The width of the breach is approximately 1/7 the axial length of the dam crest. After overtopping for 52 min, the body of the old pond accumulation dam begins to collapse. At this time, with the collapse of the local dam body of the new pond, the breaches of the two ponds form a ‘U’ shape, the breaches of the new pond then gradually expanding. There are many cracks on the surface of the dam body, and the dam body on both sides of the gully collapses. The gully widens rapidly along the transverse direction, and the collapse area increases. The emergence of this phenomenon indicates that the break in the new pond reaches the most dangerous moment. With the expansion of the breach, the flood peak caused by the tailings flows after the dam breaks.
3. After overtopping occurs for 81 min under the continuous erosion of the water flow, the tailings dam of the new pond is completely destroyed. A large volume of tailings flows within the pond, discharges as the gully is formed, and the new pond reaches its maximum breach. At this point, the new pond enters the discharge stage. After overflowing occurs for 95 min, the tailings dam of the old pond is destroyed, a large volume of tailings within the pond begins to flow out along the gully, and the expansion of the old pond’s breach reaches its maximum. At this time, the water level in the pond decreases to the lowest level, and the water head decreases to a lower level, rendering it unable to trigger a new dam-break disaster.

#### 3.3.2 Morphological evolution of dam cross-section

**Fig 9:**
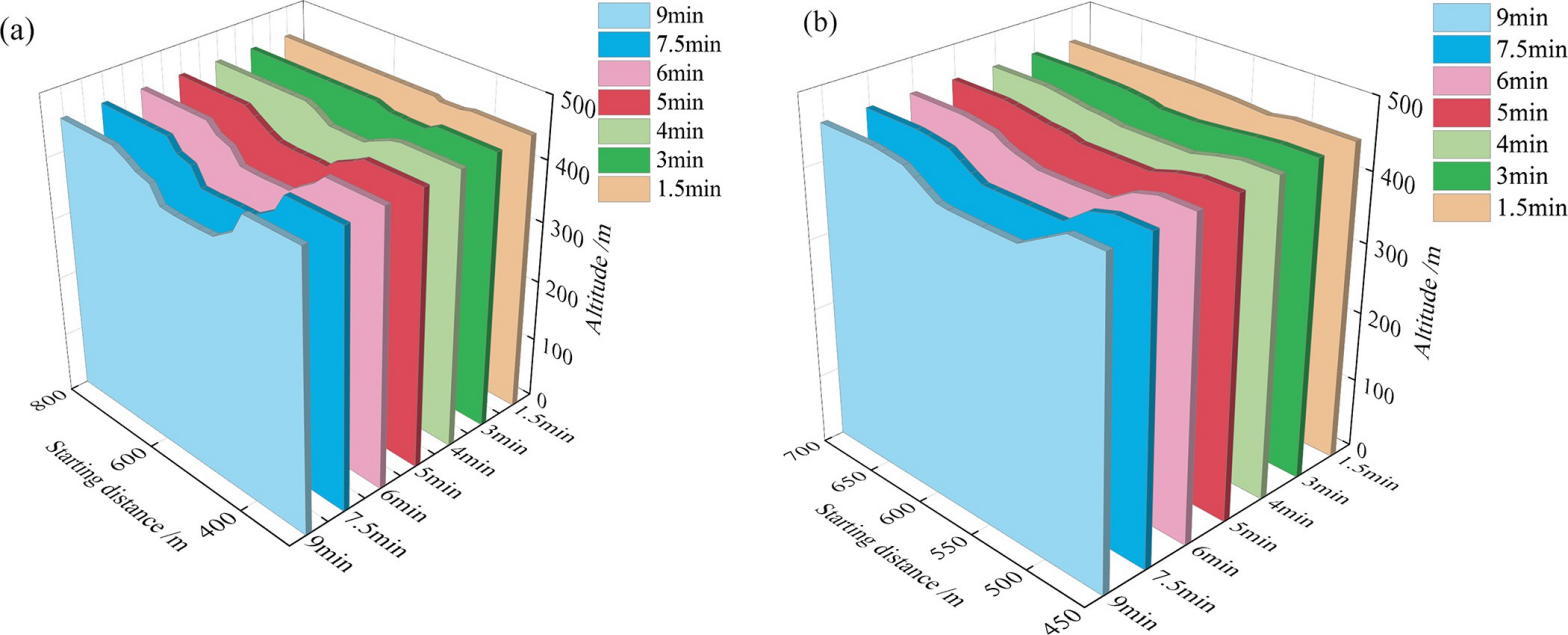
452-m section evolution process: (a) Old tailings pond; (b) New tailings pond.

The evolution process of the 452-m section after the dam break of the old and new ponds is shown in **Fig 9.** From the figure, it is evident that the 452-m section breach has a trapezoidal shape, and as time goes by, the trapezoidal shape becomes increasingly larger. At the same time, the breach of the old pond is much larger than that of the new pond in terms of both width and depth as the capacity of the old tailings pond is more significant than that of the new tailings pond, resulting in greater damage.

#### 3.3.3 Change of dam site flow

**Fig 10:**
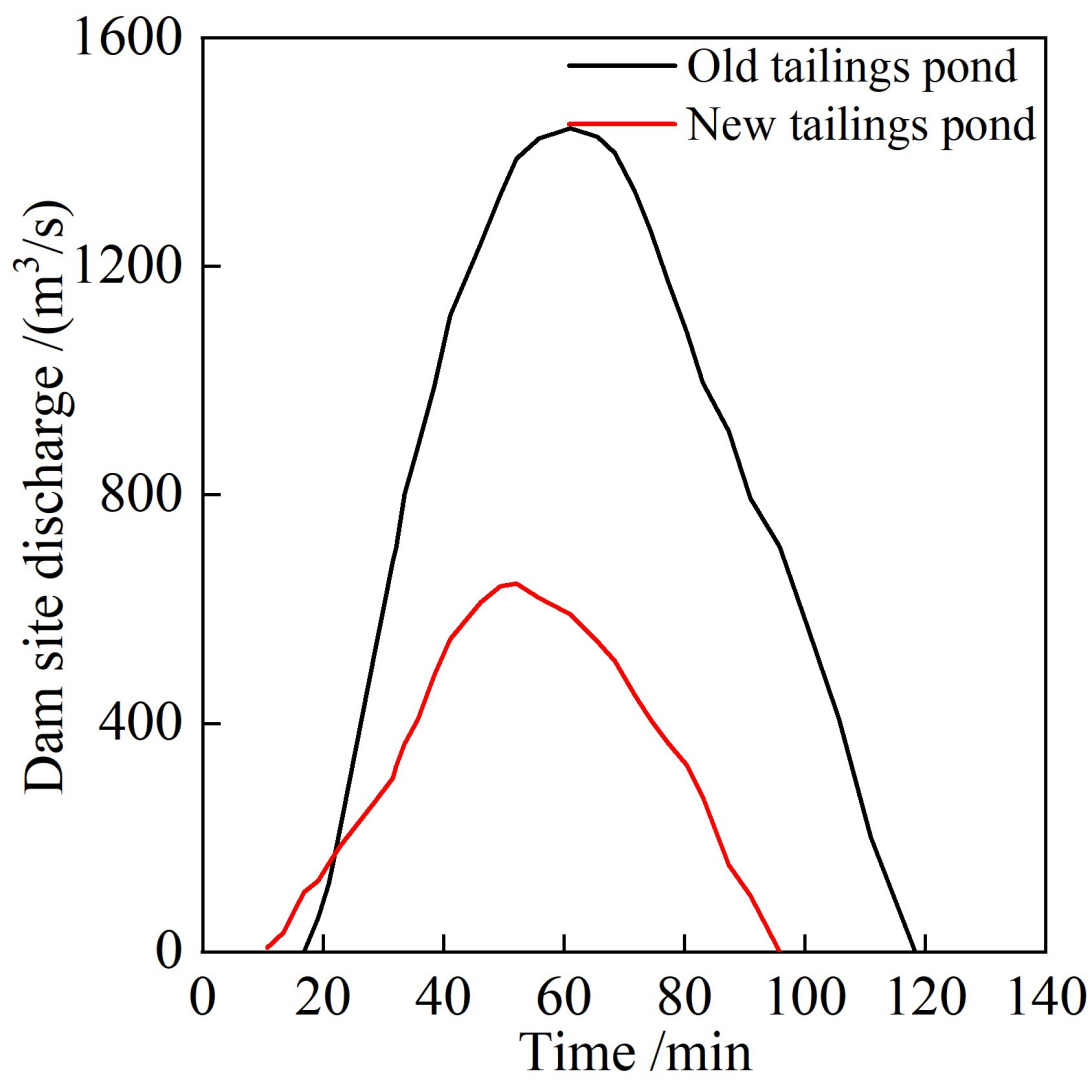
Dam section breach flow change process.

From the beginning of the flood overtopping, the time to flow to the new pond and the old pond dam foot is approximately 3.3 and 12.8 min, respectively. Moreover, the time to completely stop the flow at the dam break is approximately 98 and 130 min, respectively. The initial flow in the two ponds is small and gradually increases, two flood peaks occurring during the entire process. The peak flow in the new pond appears 41.3 min after overtopping begins, the maximum flow being approximately 665 m^3^/s. The peak flow of the old pond occurs 59 min after overtopping starts, the maximum flow being approximately 1447 m^3^/s. The peak flow of the new pond occurs before that of the old pond; however, the flow increases to the peak process, the increase being slower than that of the old pond. The storage capacity of the old pond is more significant than that of the new pond, the flood peak duration being longer than that of the new pond. Subsequently, the breach flow in the two ponds gradually decreases and tends to stabilize, the durations of the stable discharge stage being 39 and 56 min, respectively. During the flood peak period shown in the figure, there are small fluctuations in the flow of the two ponds, mainly because of the discontinuous model sand collapse at the breach during the dam-break process. Because of this collapse, the flood peak lasts for a long time, the details of which are shown in **Fig 10**.

#### 3.3.4 Section shape after dam break

**1. Section shape of dam body in the pond area**

**Fig 11:**
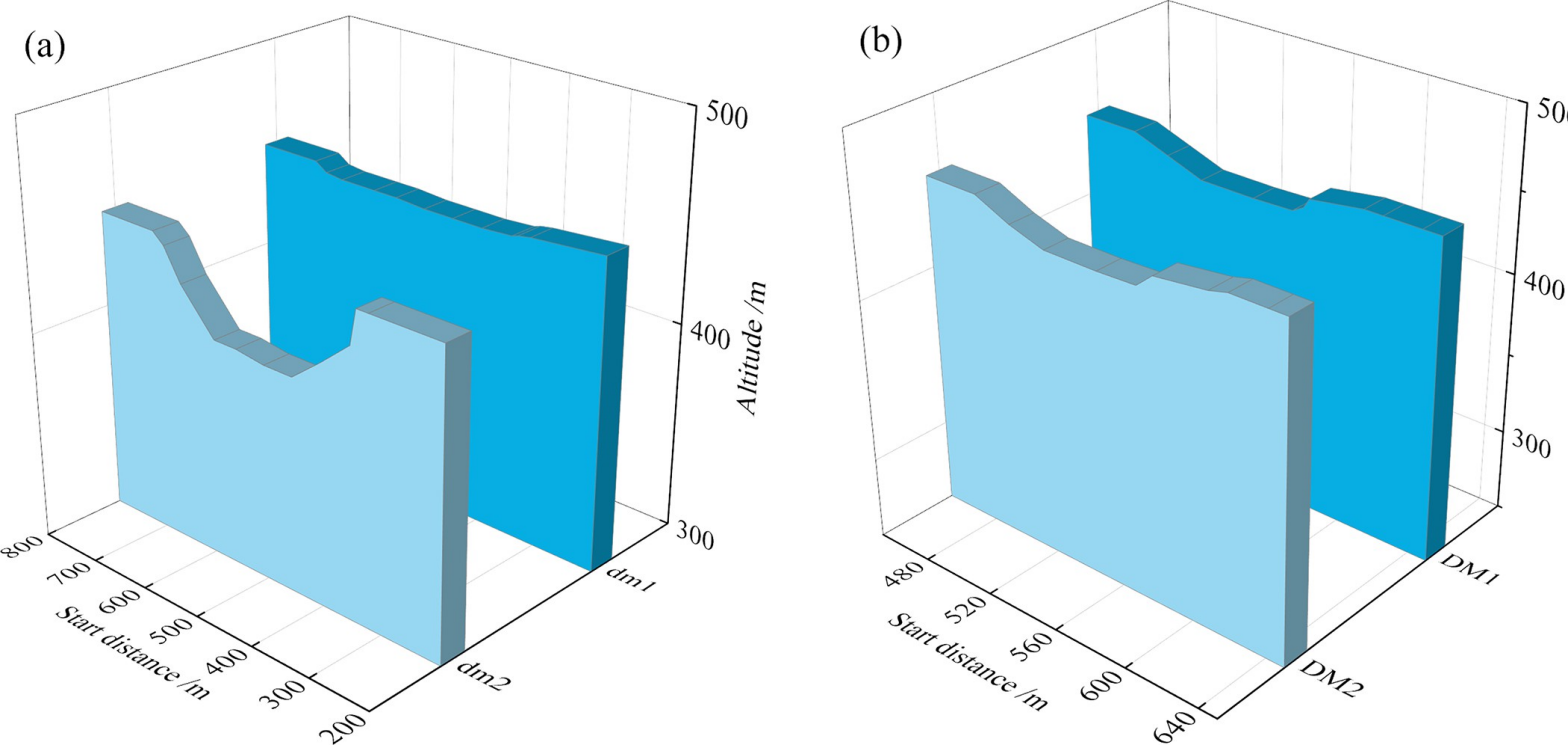
Scouring and silting form of section in pond area: (a) Old tailings pond; (b) New tailings pond.

The scouring section within the pond area is shown in **Fig 11**. The internal sections of both the old and new ponds are located in the middle of the dam. The middle water level in the pond is deeper and the sand-carrying capacity is excellent.

**2. Section shape of the accumulation dam**

**Fig 12:**
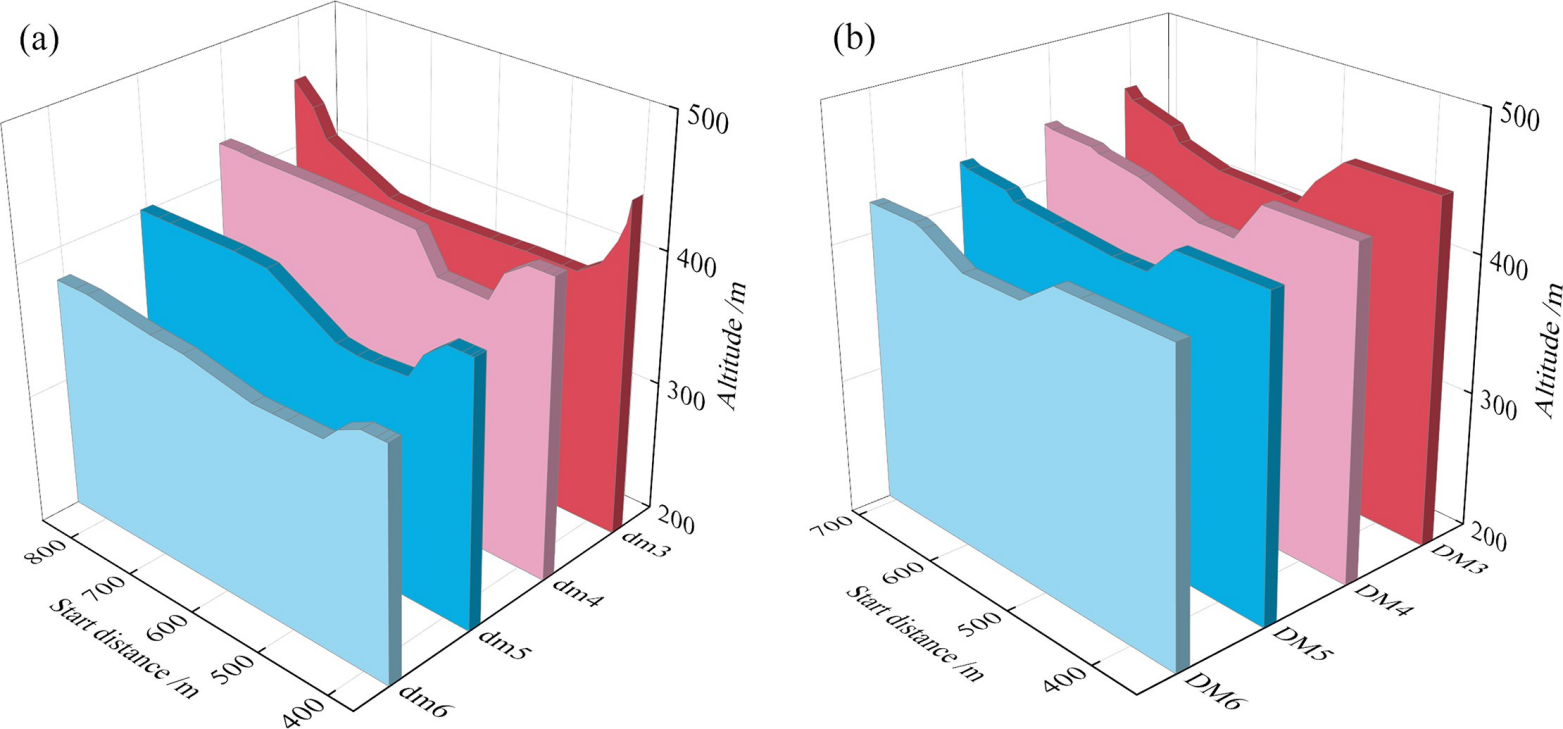
Scouring and silting form of pond accumulation dam section: (a) Old tailings pond; (b) New tailings pond.

The shape of the cross-section of the stacking dam is shown in **Fig 12**. The elevation of the **dm3** and **DM3** sections is 452 m. The maximum breach widths at the top of the dam are approximately 270 and 178 m, and the depths are approximately 57 and 40 m, respectively. With the downward movement of the location of the monitoring cross-section, the depth and spreading width of the cross-section of the new pond are greater as the elevation of the new pond stacking dam cross-section is greater than that of the old pond cross-section.

**3. Section shape of the primary dam**

**Fig 13:**
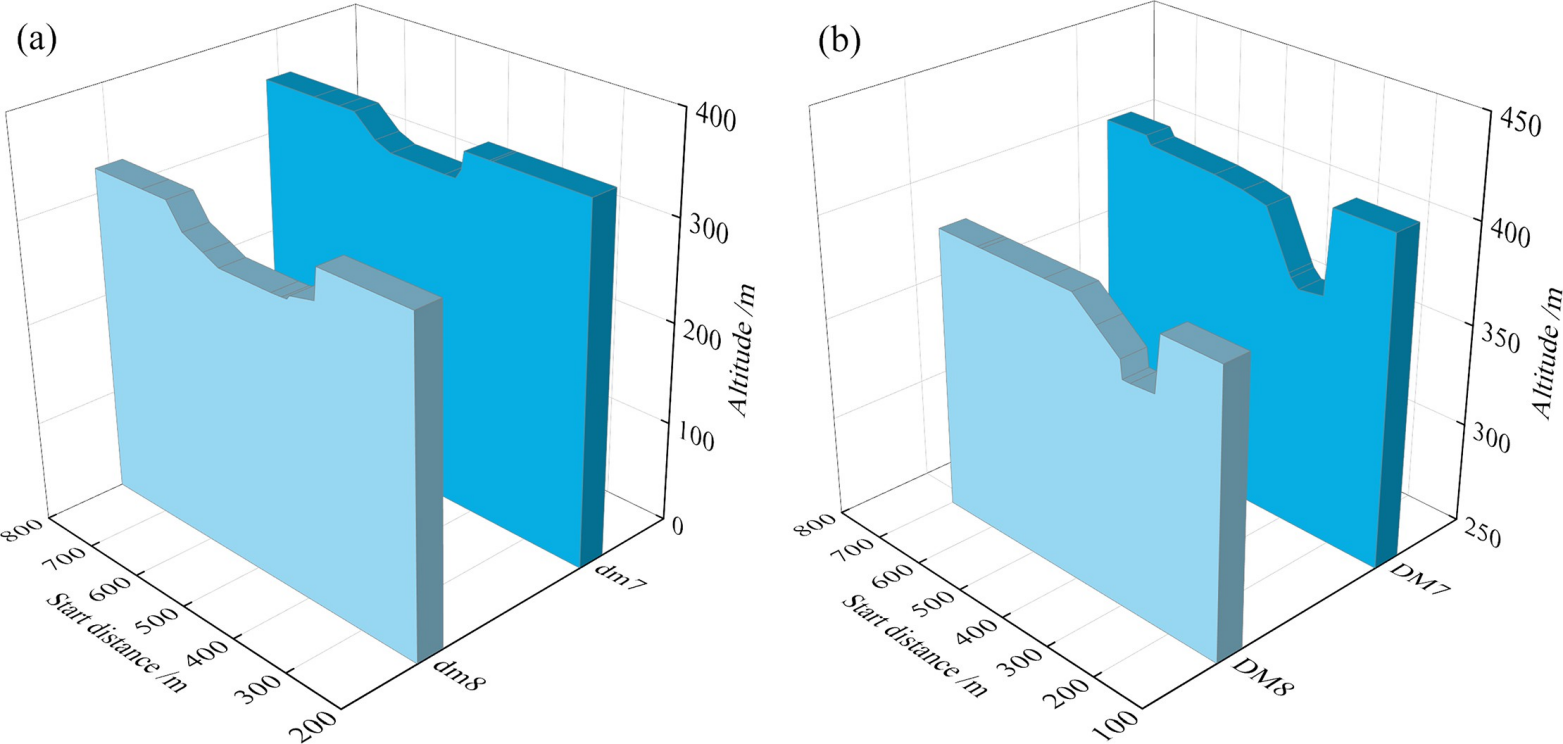
Scouring and silting form of primary dam section of pond: (a) Old tailings pond; (b) New tailings pond.

The section after the primary dam breaks is shown in **Fig 13**. The two cross-sectional breaches of the old pond are in the middle of the section, while the two cross-sectional breaches of the new pond are in the middle and right-hand sides. Because of the variable width of the primary dam of the new pond, the trend of the tailings flow changes, the flow rate of the new pond increasing slowly, the fluidity of the tailings improving, and the maximum scour widths and maximum depths of the two sections being similar.

**4. Sedimentation morphology of the downstream section**

**Fig 14:**
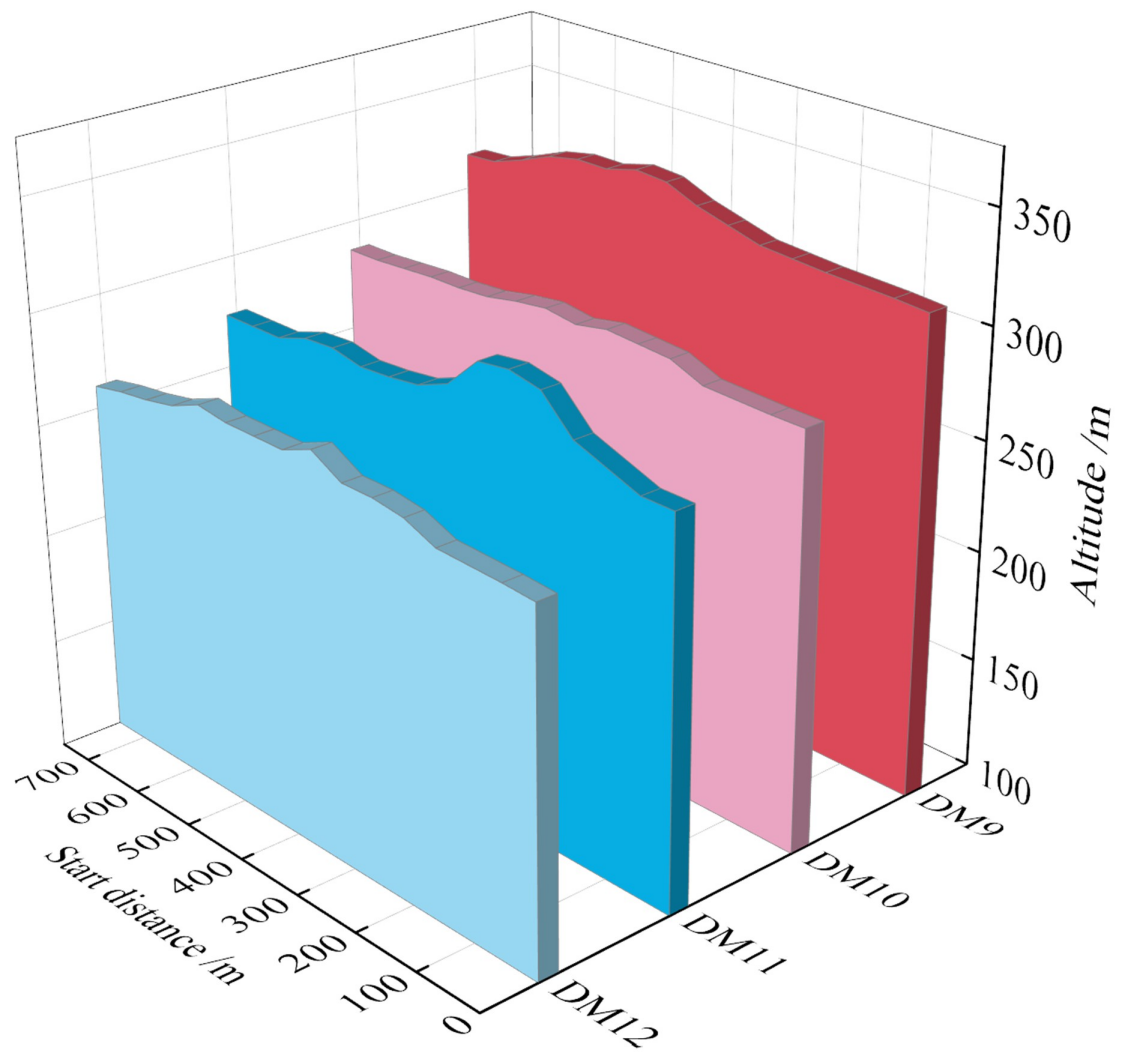
Scouring and silting form of downstream section.

Breaches of the two tailings dam overtopping model tests were conducted in the middle of the dam crest. After overtopping, the water flow exhibits a considerable decrease and high gravitational potential energy. The tailings flow rapidly downwards and continuously scour the dam body. The interaction between the two tailings dams forms a connected surface during rainfall, resulting in a long sedimentation distance downstream of the dam body. Most of the model sand flows into the reservoir, there being a small volume of model sand at the copper mine venue. The morphology of sedimentation in the downstream section of the dam after the dam break is as shown in **Fig 14**.

#### 3.3.5 Changes in downstream water level

**Fig 15:**
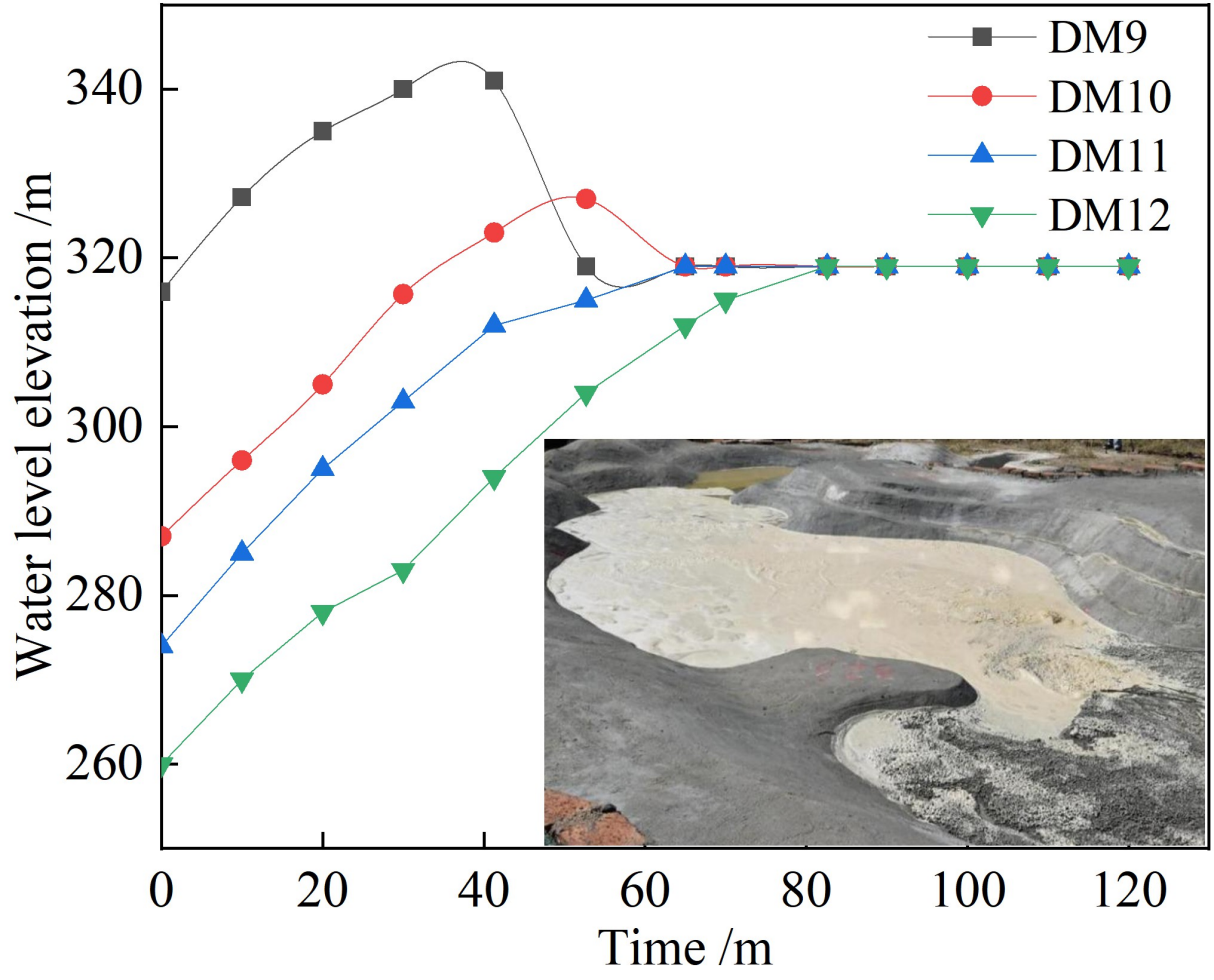
Water level process lines of different sections.

Four water level observation points were established in this study. The automatic water level tracking system monitored the water level change downstream of the dam break. From **Fig 15**, it is evident that the water level of each section downstream increases gradually with time, and finally stabilizes and remains unchanged. It takes 41.5 min for the tailing flow to reach the **DM12** section. Because the **DM12** section is in the reservoir area, the terrain of the reservoir area has a closed gully shape, the model sand is continuously silted, and the water level first rises before stabilizing and remaining unchanged. The highest flood level of the **DM12** section is 317 m, which is the same as the highest flood level of the **DM11** section.

## 4 Numerical simulation study of overtopping dam break of tailings ponds with different rainfall intensities

### 4.1 Establishment of numerical model for overtopping dam break

Considering that a physical model test requires considerable human, material, and financial resources to study the influence of rainfall intensity on the submerged range of tailings flow, a numerical simulation was used to develop the model. In this study, MIKE 21 software was used to develop a two-dimensional model. The submerged range of the overtopping dam break under different rainfall intensities was analyzed and compared with the physical model test results.

#### 4.1.1 Numerical model development

Before creating the working area generated by the grid, it was necessary to extract the elevation points in the boundary and area of the tailings dam and all downstream structures using a CAD map with elevation data, converting the data points containing the geographic location information into an XYZ format file. After map projection matching was imported into the terrain editor, the simulated *E* domain could be obtained, and the region was meshed. After interpolation, a grid file was generated using the terrain information.

**Fig 16:**
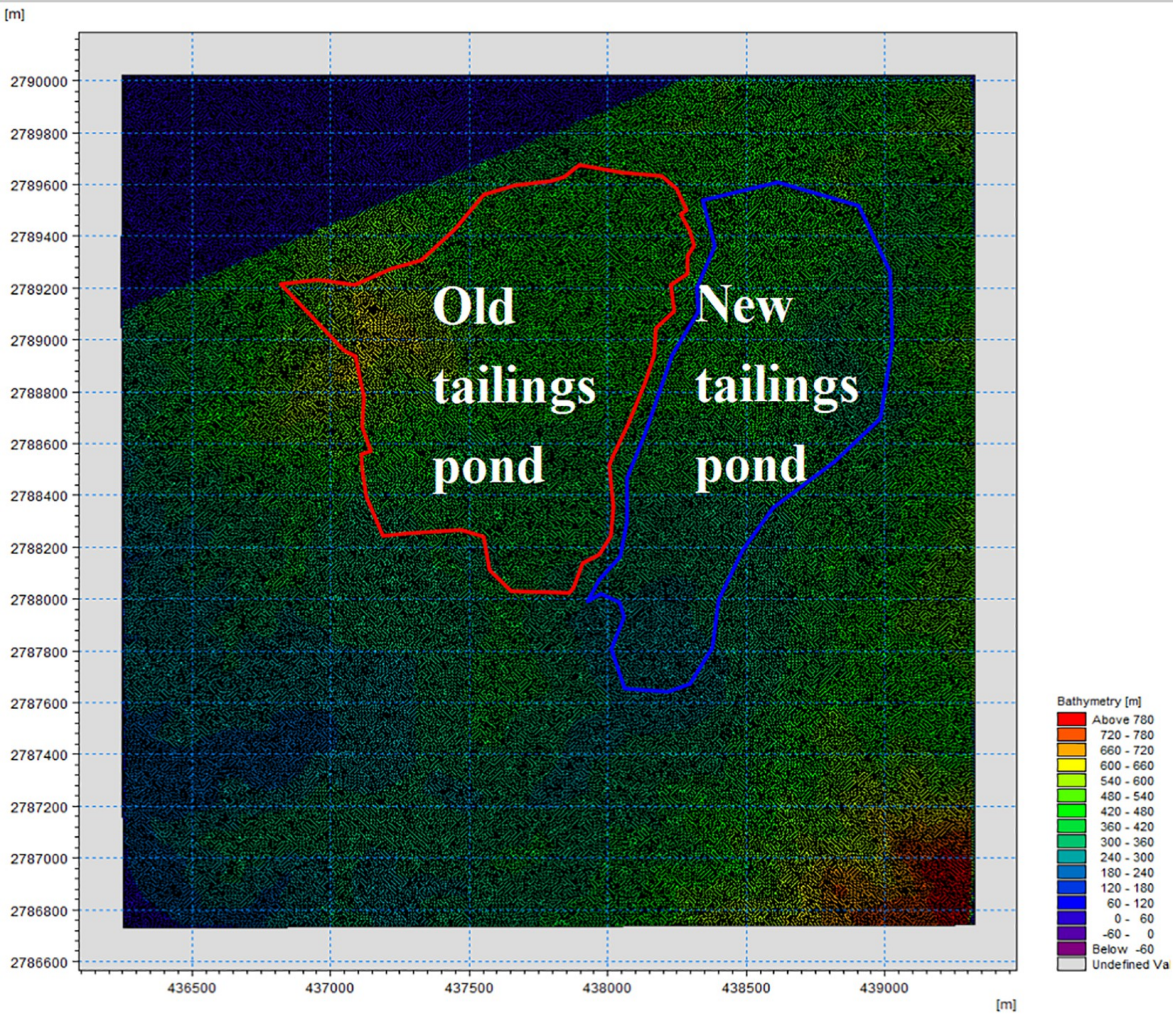
2-D model calculation grid of the old tailings pond and new tailings pond projects.

**Fig 17:**
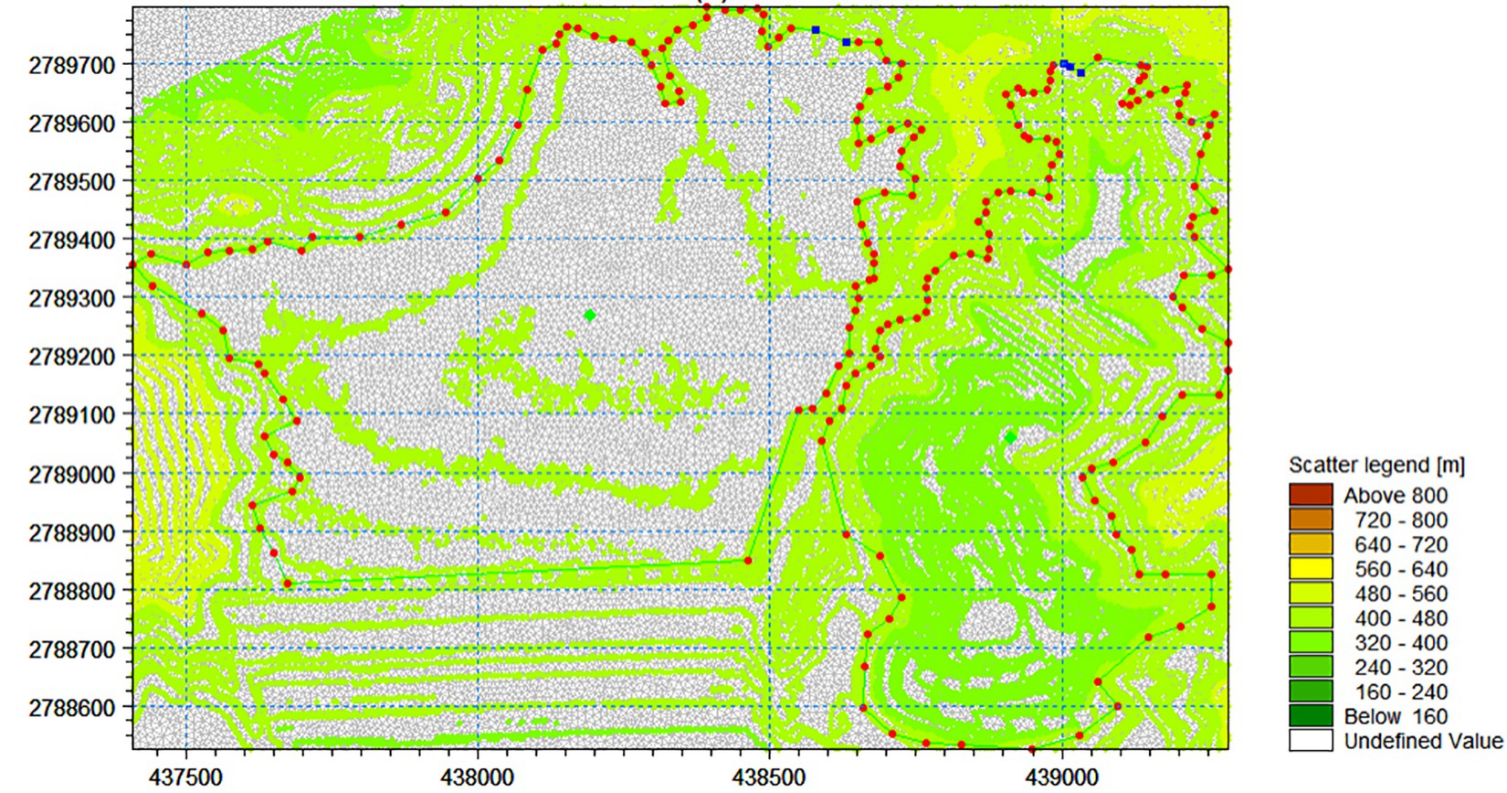
Terrain interpolation results of the old and new ponds.

1. Boundary conditions: There were two types of boundary conditions in the two-dimensional model calculation—that is, open and closed. The closed boundary in the model was the land boundary, and the open boundary was generally the water level boundary.
  - ① Closed boundary setting: In the model, the closed boundary was mostly the land boundary, and the flow velocity variable on the boundary was 0.
  - ② Open boundary setting: The upper boundary was the pond water level *dfso* file, and the lower boundary was the River water level *dfso* file.
2. Grid division: An unstructured triangle was used to divide the area affected by the tailings ponds, as it has an excellent complex area and can simulate the natural boundary and complex underwater terrain well; thus, the boundary simulation accuracy is higher. The calculation range of this two-dimensional model was located primarily in the pond area and downstream of the old and new ponds. The calculated area was 10.02 km^2^. An irregular triangular mesh was used to obtain 150,000 meshes, the mesh being used for simultaneous simulation of the old and new ponds at the same time. The grid was divided based on the terrain data of the influence area of the tailings dam, as shown in **Fig 16**.
3. Terrain interpolation: Based on the layout scheme and current situation of the primary dam and accumulation dam of the old and new ponds, the topography of the old and new ponds was processed, and the above-mentioned completed grid areas were interpolated. Different elevation sections are displayed in different colors, the resulting plane graphics of which are shown in **Fig 17**.

#### 4.1.2 Selection of model parameters

In this numerical simulation, MIKE21 was used to simulate the overtopping dam break of two tailings pond projects simultaneously, a two-dimensional dam-break evolution model being developed. In the MIKE2l model, the parameters to be set were the calculation time and calculation step, bottom friction force, wind field, and eddy viscosity.

1. The total simulation time for the simultaneous overtopping and breach of the two tailings ponds was set to 2 h, or 7,200 s.
2. The calculation step size was set to 36 s to meet the stability requirements of the model.
3. The simulation did not consider water evaporation, the net rainfall intensity being consistent with the physical model, that is, 2.35 mm/min;
4. The vortex viscosity parameter was calculated using the Smagorinsky equation using a value of 0.28 m^2^/s;
5. Owing to the small impact of the wind field on the dam-break simulation, the wind field setting was ignored in this calculation.
6. This simulation used Manning’s coefficient *M*_u_ to represent the bottom friction force, with *M*_u_ ranging from 3.2–32;
7. The calculation in this model did not consider the influence of the Coriolis force.

### 4.2 Analysis of numerical simulation results of overtopping dam break

**Fig 18:**
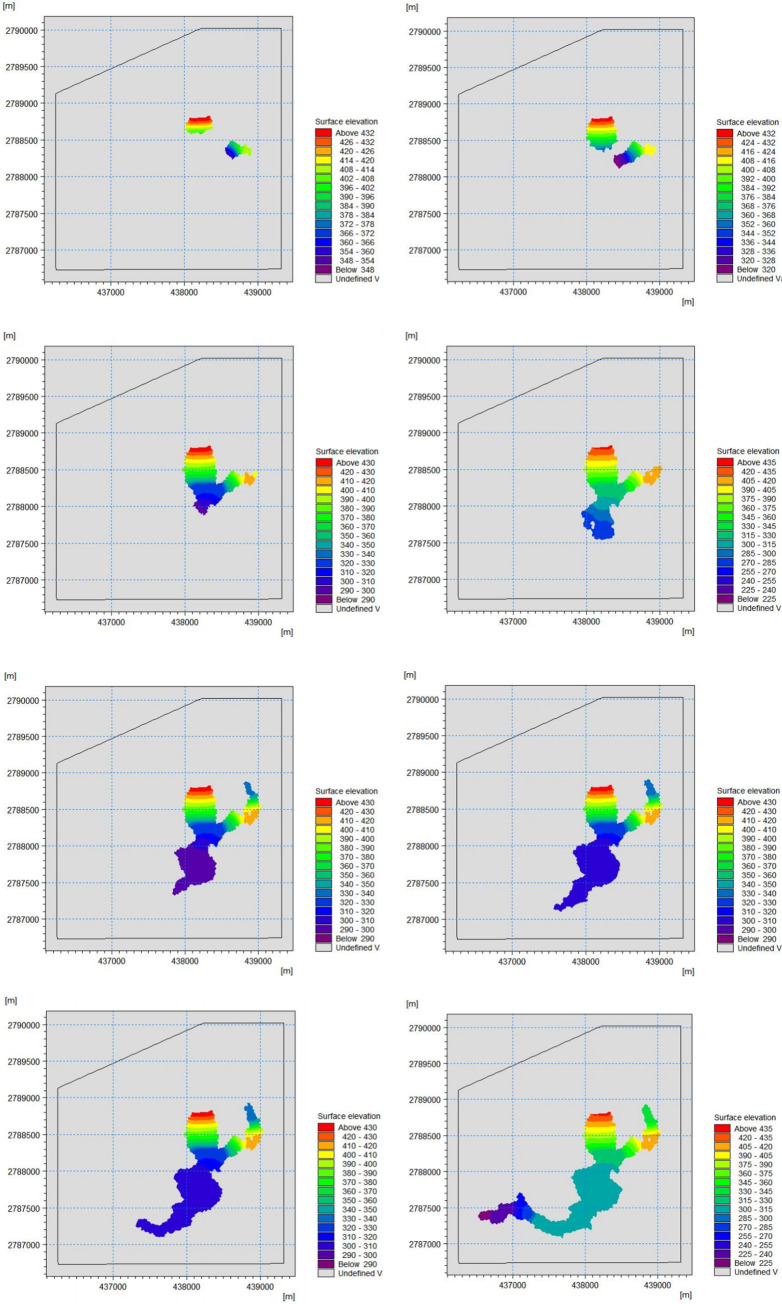
Numerical simulation of the impact area evolution of the dam break. (a) 2 min, (b) 5 min, (c) 10 min, (d) 20 min, (e) 30 min, (f) 60 min, (g) 90 min, (h) 120 min.

**Fig 19:**
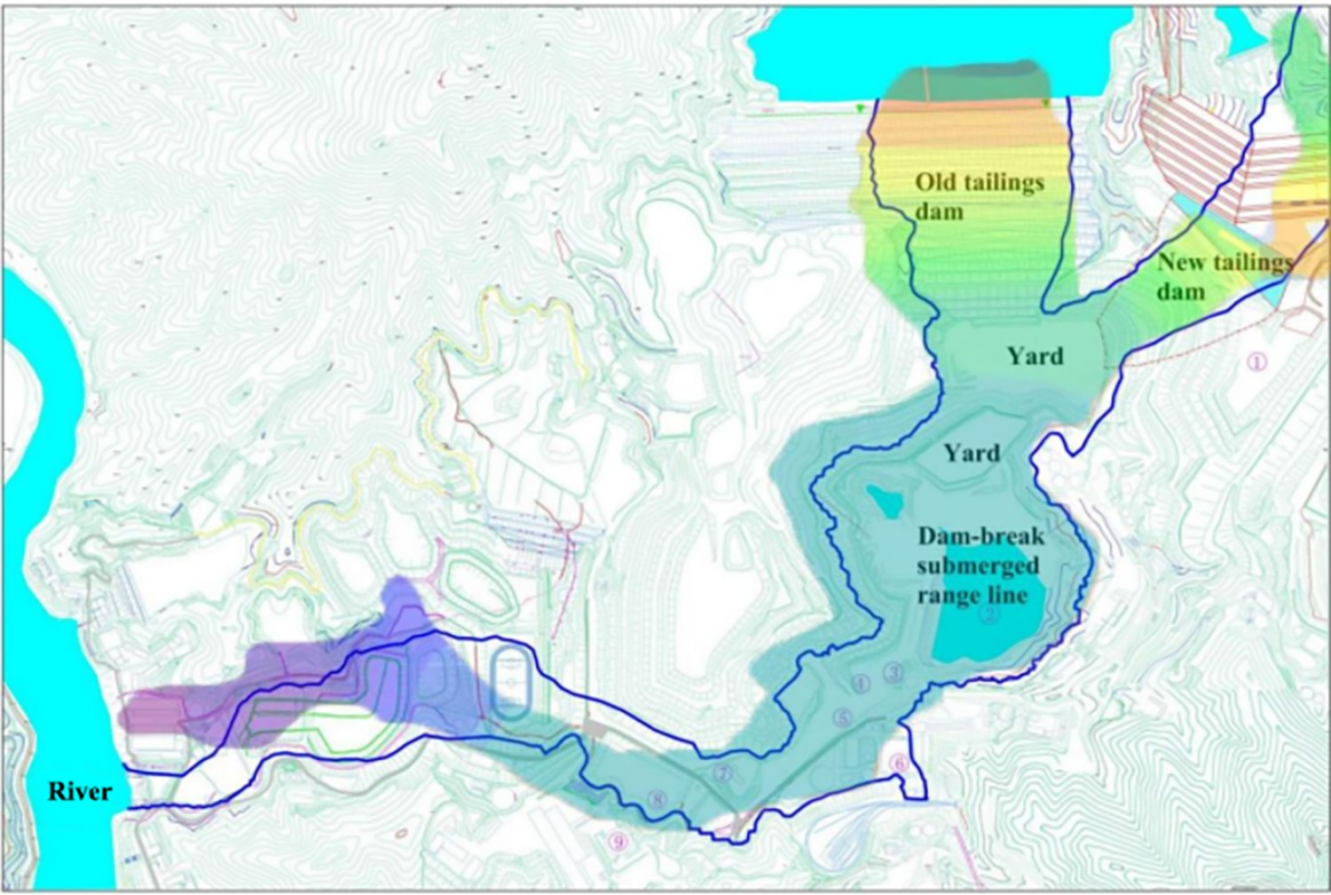
Comparison between the physical test and numerical simulation submerged area.

This numerical simulation analyzed the influence of the submerged range after the dam break of the tailings pond simultaneously, ignoring the order of the dam break between the old and new ponds and considering the dam breaks to be simultaneous. The evolution of the submerged range of the simulation results under these working conditions is shown in **Fig 18**. Cloud map analysis compared the numerical simulation results of the submerged range after the dam break with those of the submerged range of the model test. The submerged range obtained from the numerical simulation is slightly more extensive. The state of each facility obtained from the numerical simulation is basically the same as that of the physical model test results, and facilities 2–8 are all within the influence of the dam failure. Facility 6 in the numerical simulation was not completely inundated, but was still affected by the dam failure. The scope of the impact of the inundation is shown in **Fig 19.**

### 4.3 Analysis of impact of rainfall intensity on the inundation range of overtopping dam break

**Fig 20:**
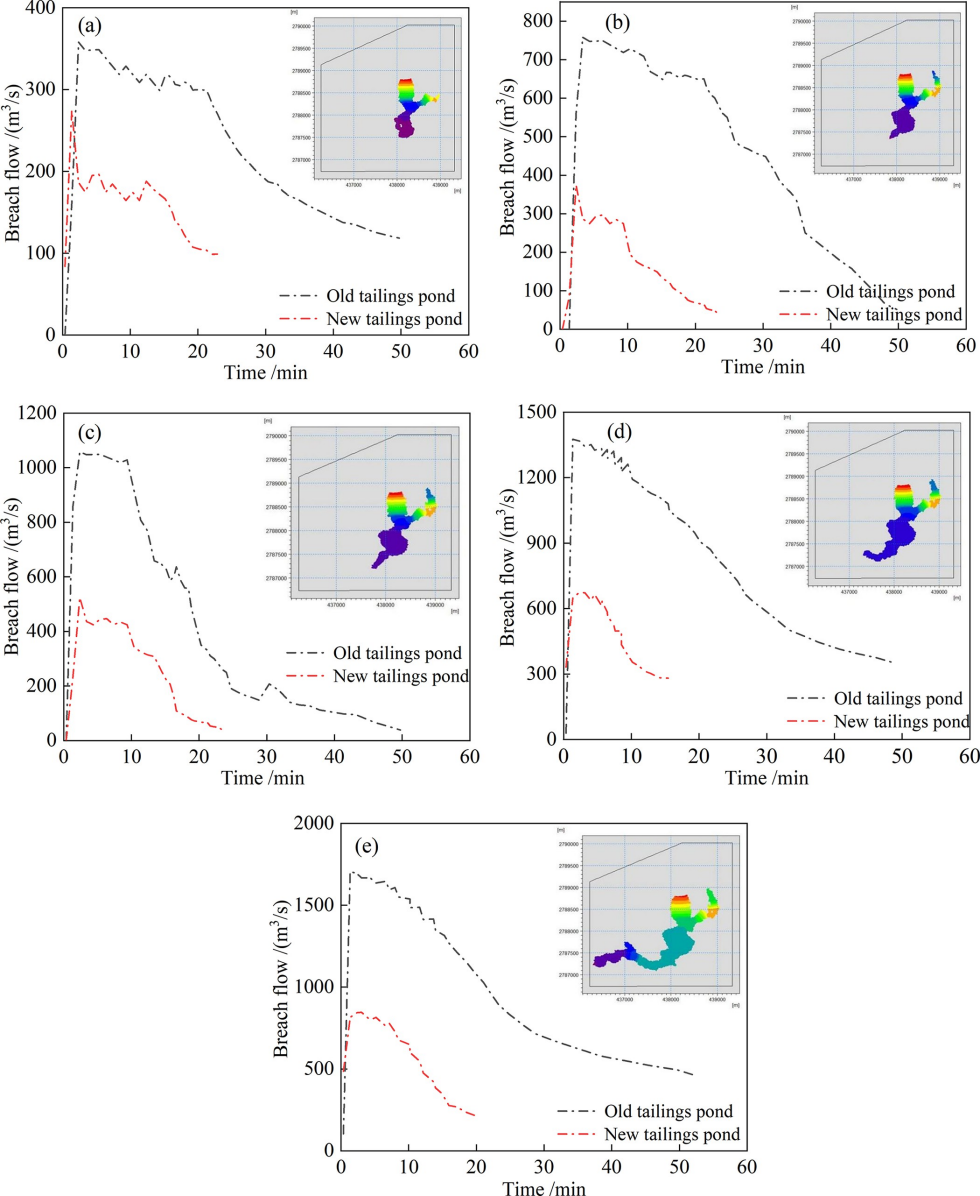
The breach flow process at different rainfall intensities. (a) Condition 1, (b) Condition 2, (c) Condition 3, (d) Condition 4, (e) Condition 5.

To safely protect downstream residents and structures, it is necessary to analyze the influence of different rainfall intensities on the submerged area. We simulated five different rainfall intensities—that is, 1.6, 1.8, 2.0, 2.2, and 2.4 mm/min—as well as other parameters. The breach flow process lines and final inundation ranges of the two tailings ponds under different rainfall intensities are shown in **Fig 20**. With an increase in rainfall intensity, the breach flood peak flow of the old pond appears later; however, the breach flood peak flow becomes increasingly substantial, and the inundation range increases.

**Fig 21:**
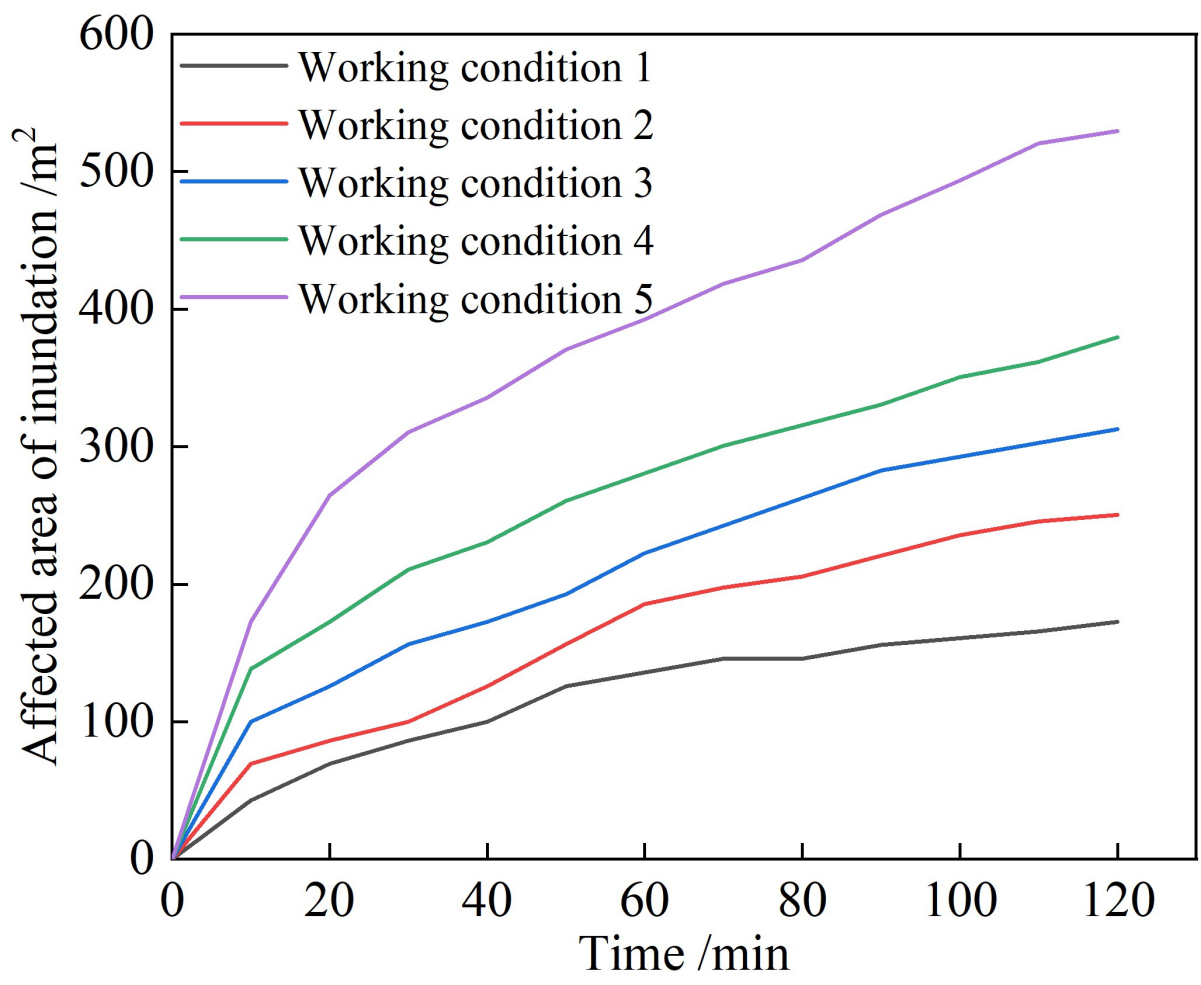
Impact area rule of water and sand mixture of the tailings dam break.

**Fig 22:**
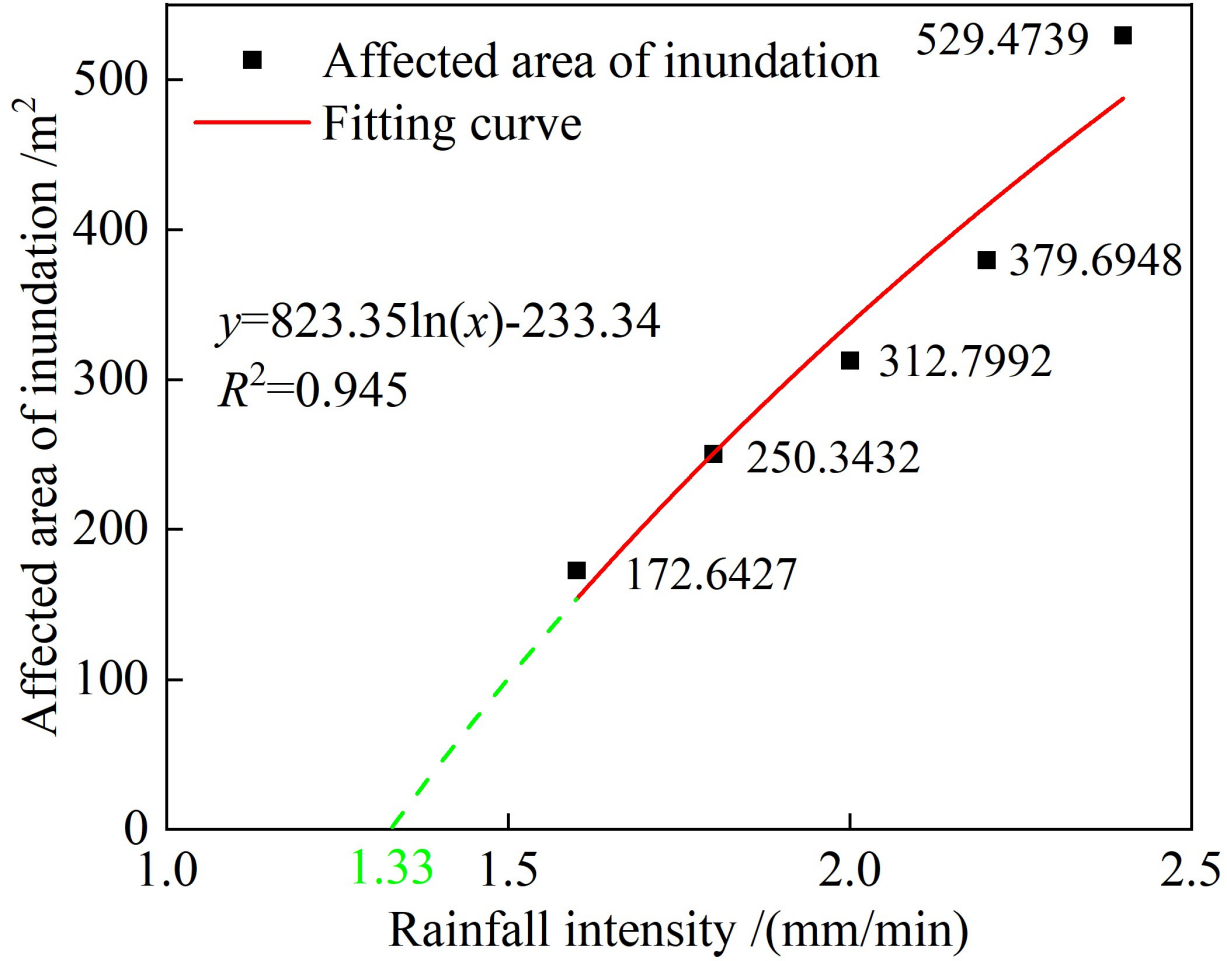
Fitting curve of the rainfall intensity and submerged area.

Based Impact area rule of water and sand mixture of the tailings dam break shown in **Fig 21**, the influence area of the tailings flow submergence could be obtained using Image-Pro Plus 6.0 digital image processing software. To ensure the reliability and accuracy of the results, the quantitative length was set to 10 m. With an increase in rainfall time, the influence area of the water-sand mixture increases rapidly, but the increase is slow in the later stages of the dam break. The rainfall intensity and submerged ranges can be numerically fitted, the fitting curve being as shown in **Fig 22**. The fitting relationship between the submerged range and rainfall intensity satisfies the logarithmic function relationship y = 825.35ln(x)−233.34. When the rainfall intensity is < 1.33 mm/min, the submerged influence area is 0—in other words, overtopping dam breaks do not occur. The submerged influence area gradually increases as the rainfall intensity increases, but the rate of increase decreases. The fitting curve is consistent with the actual situation.

### 4.4 Analysis of the impact of protective measures on the inundation range of overtopping dam break

Based on the above model test and numerical simulation results, the inundation range of the tailings pond was considerable downstream of the simultaneous dam break. For the safety of downstream residents and structures, setting up a retaining dam downstream is an effective safety measure. However, the dam height and location affect the protective effect of the retaining dam. The following section analyzes the protective effect in terms of both dam height and location. The check dam was assumed to be rigid. This model was based on the above numerical model but added a retaining dam to the structure module with a rainfall intensity of 2.35 mm/min and rainfall duration of 2 h.

#### 4.4.1 Impact of retaining dam location

**Fig 23:**
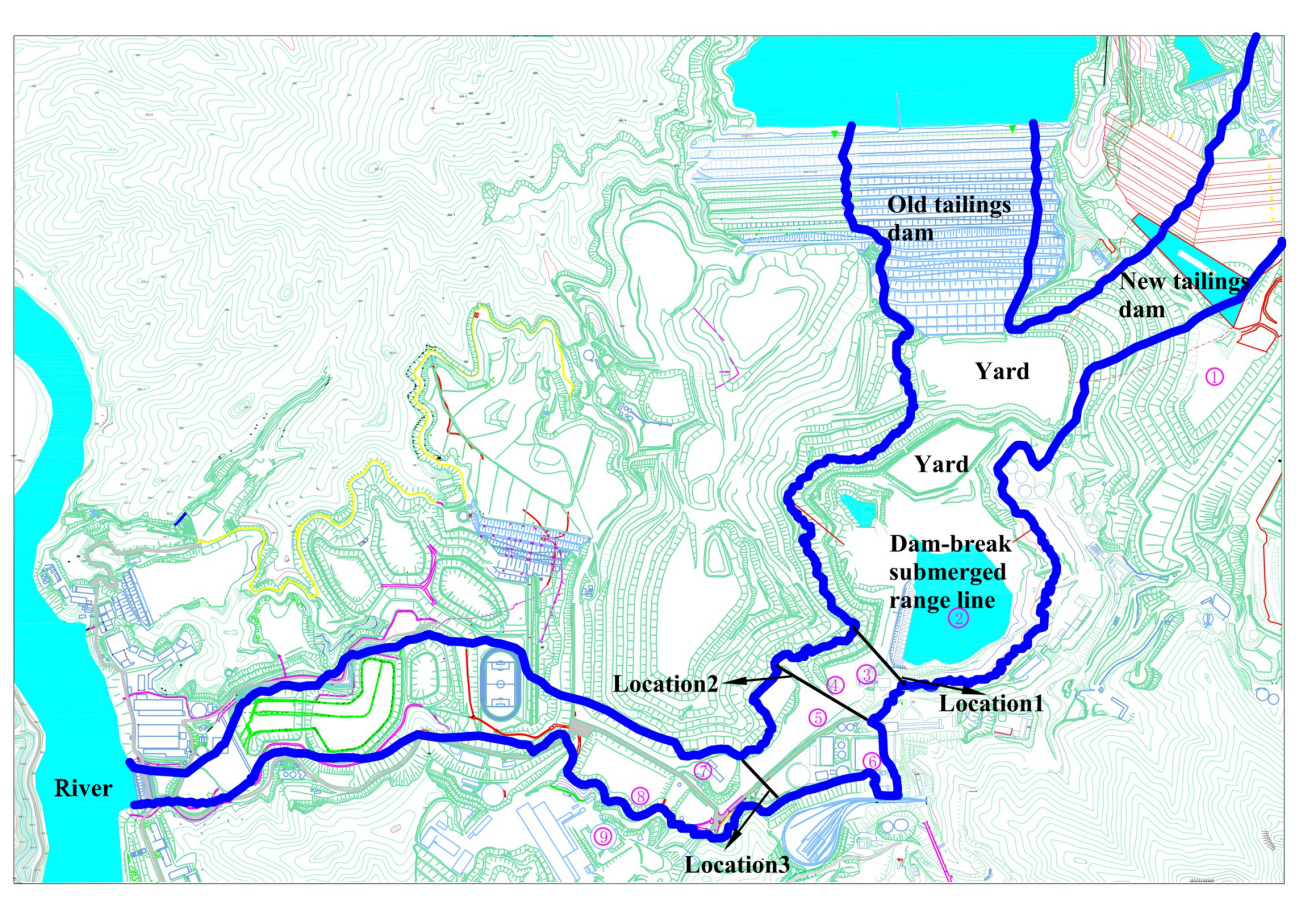
Location of the three retaining dams.

The location of the retaining dam can have a particular influence on the submerged range of the tailings mixture. As the submerged range of tailings flows downstream, obtained by comparing the above physical model test and numerical simulation, it is evident that the tailings flow submerges most of the downstream structures. Thus, it is essential to set up a downstream retaining dam to protect the downstream structures. From an overall submerged range perspective, the aim is to protect all structures after structure ⑧. Consequently, three retaining dams were set up at different locations downstream of the tailings ponds. We set up **Location 1** next to structure ②, which could intercept most of the tailings flow, although the tailings flow velocity at this location is large; we set **Location 2** below structure ④, sandwiched between **Location 1** and **Location 3**, the width of the dam being the longest. **Location 3** was set below structure ⑤, which was at the mouth of the dam; its width being the smallest. The locations of the three retaining dams are shown in **Fig 23**.

**Fig 24:**
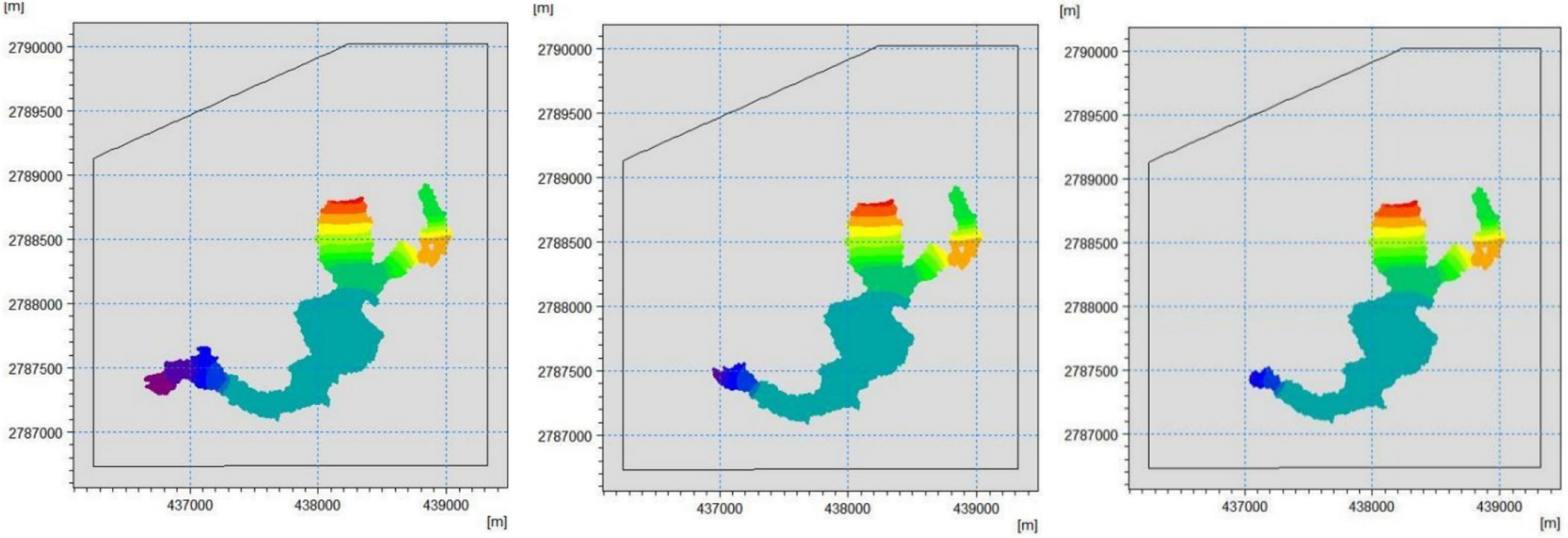
Map of the submerged range of the three retaining dams: (a) Location 1; (b) Location 2; (c) Location 3.

The retaining height of the three locations was 8 m, and the interception effect of retaining dams of the same height but different locations on the tailings mixture was investigated. **Fig 24** shows that the area affected by the water-sand mixture of the retaining dam at **Location 1** is approximately 440.3376 m^2^, which is not much different from the 477.6808 m2 without the retaining dam. The tailings mixture at **Location 1** has a fast flow velocity, high water level, and poor interception effect, whereas the impact area of the water-sand mixture of the retaining dam at **Location 2** is approximately 398.9113 m^2^, the intercepted inundation area accounting for 16.49% of the original inundation area without the retaining dam. The interception effect is better than that at **Location 1** from the perspective of the inundation area of the tailings flow. Because of the width of **Dam 2**, the tailings flow extends to both sides, increasing the inundation area, the impact area of the water-sand mixture of the retaining dam at **Location 3** being approximately 359.7706 m^2^, reducing the inundation area by 24.67% of the original non-retaining dam inundation area. The interception effect is better than that at **Locations 1** and **2**, and the width of the retaining dam at **Location 3** is the smallest, resulting in lower economic costs. From the above analysis, the location of the retaining dam at **Location 3** is more in line with engineering practice.

#### 4.4.2 Impact of retaining dam height

**Fig 25:**
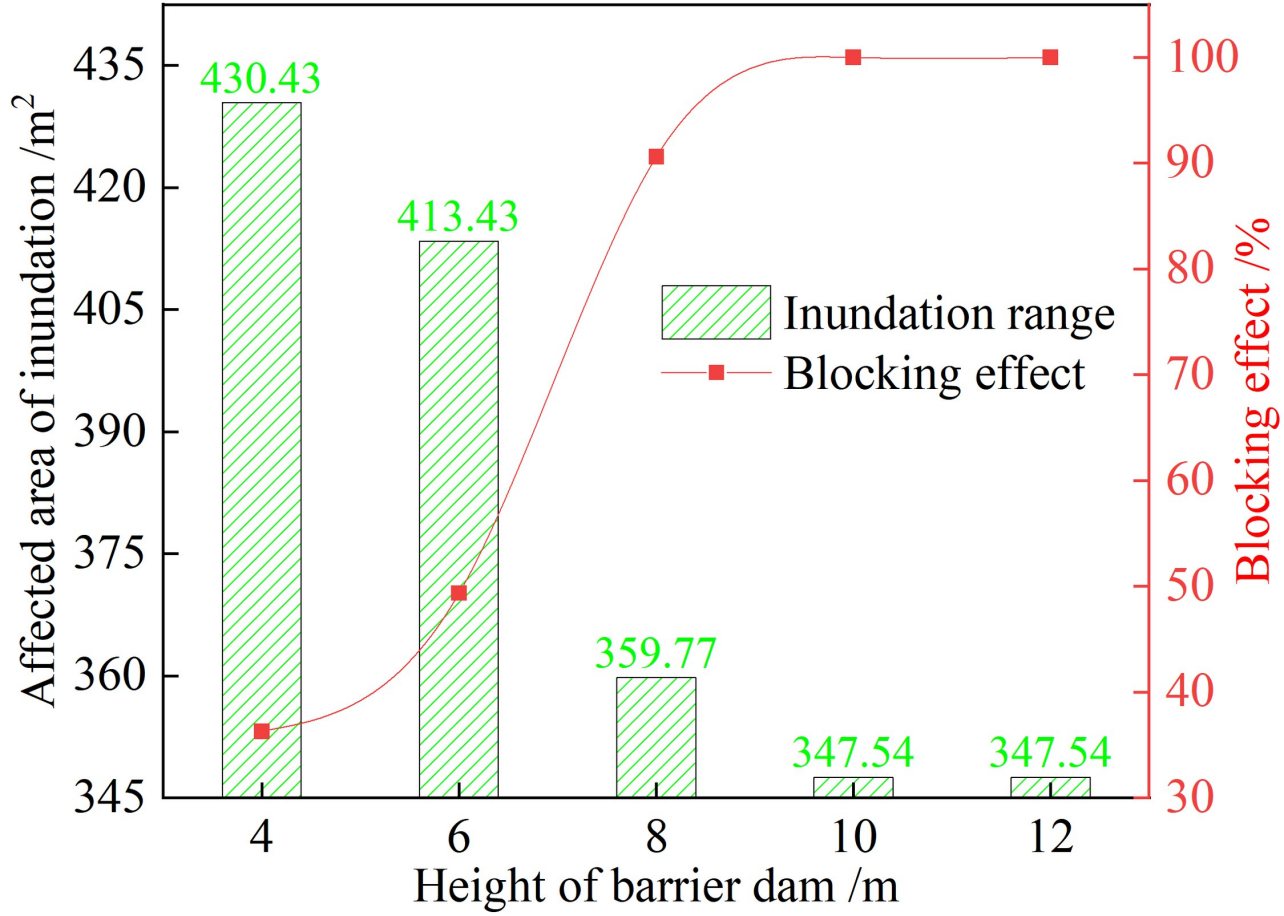
Interception effect of the retaining dam at different heights at Location 3.

Based on the above analysis, setting up a retaining dam at **Location 3** was the most suitable. The impact of different retaining dam heights—that is, 4, 6, 8, 10, and 12 m—on the inundation range of the new and old tailings ponds due to simultaneous overtopping and dam breaks is shown in **Fig 25**. It is evident that with an increase in dam height, the submerged influence area of the dam at **Location 3** decreases, but at 10 m, the interception effect does not change, suggesting the 10 m blocking effect of the dam at **Location 3** to be the best. It is highly effective in intercepting the discharge of tailings and in reducing the property loss of downstream residents and structures.

## 5 Conclusions

This study is based on an old tailings pond that is about to be put out of service and the proposed new tailings pond next to it. Field investigations, laboratory tests, model tests, and numerical simulations were combined to study the inundation range of the new and old tailing ponds with simultaneous overtopping dam breaks under actual terrain conditions. First, fine-grained tailings and expanded perlite were selected as the model sand materials, and the appropriate model sand ratio was determined through laboratory tests. Second, the two tailings ponds were tested (at a scale of 1:200), for flood overtopping and simultaneous dam breaks under a rainfall intensity of 2.35 mm/min. The dam break, flow, section morphology evolution, submerged elevation, and range were analyzed. Finally, a numerical model was developed using MIKE 21 to simulate the simultaneous overtopping and collapse of the new and old tailings ponds. The impact of rainfall intensity on their inundation range was analyzed. By adding a blocking dam to the structural module, the impact of the location and height of the retaining dam on the inundation range of the overtopping dam could be determined.

1. Based on many flume tests, a suitable model sand ratio was the 1.5:1 ratio of fine-grained tailings to expanded perlite. The flow velocity scale was similar to the starting velocity scale, which met the starting similarity requirements. The basic physical and mechanical parameters of the model and prototype sands were obtained using indoor geotechnical tests. The results showed that the model sand and prototype sand met the model similarity conditions, and that the model sand could be applied to the physical model test.

Based on the actual engineering situation and test site conditions, the actual terrain was restored at a scale of 1:200, and the overtopping dam break tests of the new and old tailings ponds were conducted simultaneously when the rainfall intensity was 2.35 mm/min. The peak flow of the new pond was approximately 665 m^3^/s, the peak flow of the old pond being approximately 1447 m^3^/s. The burst flood peak lasted for 9.8 and 21 min, respectively, before the breach flow gradually decreased and stabilized, the durations of the stable discharge stages being 28 and 76 min, respectively. After the dam breaks in the old and new ponds, the top section of the dam exhibited gradual trapezoidal expansion; the water level of each section downstream increased gradually over time before stabilizing at a water level of 317 m. The final submerged range of the tailings flow reached River.

The model test and numerical simulation results of the inundation range of the new and old tailings ponds with simultaneous overtopping and dam breakage were consistent, the simulations verified the reliability of the parameter selection in the numerical models. By simulating the simultaneous dam break of two tailings ponds under different rainfall intensities, it was evident that the relationship between rainfall intensity and the inundation range of the dam break followed a logarithmic function form. Based on the above model, the results showed that a height of 10 m at **Location 3** in setting up a retaining dam was the most suitable, as it could completely intercept the tailings flow and protect the safety of downstream residents and structures.
